# The underlying neurobiology of key functional domains in young people with mood and anxiety disorders: a systematic review

**DOI:** 10.1186/s12888-016-0852-3

**Published:** 2016-05-23

**Authors:** Frank Iorfino, Ian B. Hickie, Rico S. C. Lee, Jim Lagopoulos, Daniel F. Hermens

**Affiliations:** Clinical Research Unit, Brain and Mind Centre, University of Sydney, 94 Mallet Street, Camperdown, NSW 2050 Australia

**Keywords:** Depression, Anxiety, Bipolar, Functional outcomes, Biomarkers, Neurobiology, Neuropsychology, Personalised psychiatry

## Abstract

**Background:**

Mood and anxiety disorders are leading causes of disability and mortality, due largely to their onset during adolescence and young adulthood and broader impact on functioning. Key factors that are associated with disability and these disorders in young people are social and economic participation (e.g. education, employment), physical health, suicide and self-harm behaviours, and alcohol and substance use. A better understanding of the objective markers (i.e. neurobiological parameters) associated with these factors is important for the development of effective early interventions that reduce the impact of disability and illness persistence.

**Methods:**

We systematically reviewed the literature for neurobiological parameters (i.e. neuropsychology, neuroimaging, sleep-wake and circadian biology, neurophysiology and metabolic measures) associated with functional domains in young people (12 to 30 years) with mood and/or anxiety disorders.

**Results:**

Of the one hundred and thirty-four studies selected, 7.6 % investigated social and economic participation, 2.1 % physical health, 15.3 % suicide and self-harm behaviours, 6.9 % alcohol and substance use, whereas the majority (68.1 %) focussed on clinical syndrome.

**Conclusions:**

Despite the predominance of studies that solely examine the clinical syndrome of young people the literature also provides evidence of distinct associations among objective measures (indexing various aspects of brain circuitry) and other functional domains. We suggest that a shift in focus towards characterising the mechanisms that underlie and/or mediate multiple functional domains will optimise personalised interventions and improve illness trajectories.

## Background

Depression and anxiety are associated with the greatest burden of disease of all neurological, psychiatric and substance use disorders [1]. The early onset of psychiatric illness plays a key role in such disability with approximately 75 % of these disorders occurring before the age of 25 years [2]. Despite this, our current capacity to provide tailored early interventions and prevent the progression of illness or slow the pathway to disability is lacking [3]. Whilst, some specific diagnoses have been successfully treated with certain interventions (e.g. CBT for social anxiety disorder; [4]), there are limitations to the optimal treatment for unipolar, bipolar and comorbid mood disorders. This is particularly evident for those with emerging illnesses who often experience mixed states and/or subthreshold symptoms [5]. Since these mood states typically arise during adolescence and young adulthood, a period of critical brain development and functional independence, the impact of illness can lead to greater disability and worse illness outcomes [6–8]. Thus, the search for objective markers of early risk states with predicative capacity in regards to disability and mortality requires rigorous investigation so that appropriate interventions can be trialled and delivered as early as possible to reduce the impact of disability [3, 9].

Traditionally, there has been a focus on the ‘clinical syndrome’ defined as identifying distinct clinical categories or disorders (based on ICD or DSM diagnostic criteria) with specific thresholds and the impact of these on functioning. However, even at a subthreshold symptom level significant contributors to disability and mortality include social and economic disability [10, 11], poor physical health (e.g. diabetes) [12], high suicide and self-harm behaviours [10, 13–15], and risky alcohol and substance use [16, 17]. Given the clinical impact of these factors for young people with emerging mood and anxiety disorders, we have identified them as key functional domains that, we argue, should be the focus of targeted personalised assessment and intervention. Although the term ‘functional domain’ has traditionally, often referred to outcomes relating to occupational (i.e. employment and education) status, here, we use the term to include other key factors that have significant (often concomitant) impacts on levels of functioning in young people. These domains largely align with the framework provided by the *‘International classification of functioning, disability and health’* [18] for conceptualising health and health related states. These include: (i) social and economic participation (i.e. engagement and stability in employment, education and social relationships); (ii) physical health; (iii) suicide and self-harm behaviours; (iv) alcohol and substance use; and (v) clinical syndrome (i.e. diagnostic category, stage of illness and severity of symptoms [3]). These domains are priority areas for service models in Australia (e.g. *headspace* [19, 20]), which recognise the need for early interventions that aim to target specific outcomes associated with illness persistence and greater disability [21]. Importantly, a focus on these five domains recognises the need to evaluate multiple (often interacting) aspects of an individual to better characterise their specific phenotype and, as a result, attempt to predict their potential illness trajectory.

To overcome some of the limitations associated with current diagnostic approaches that link poorly to neurobiological risk factors or patterns of treatment response it is important to characterise the neurobiology that may underlie or mediate observable functional impairment(s) [9, 22]. This emphasises the need to focus on the four remaining functional domains in addition to the traditional focus on the clinical syndrome to optimise personalised interventions. Models of psychopathology suggest that breakdowns in common brain circuits involved in cognition and behaviour are responsible for the development of psychopathology and general dysfunction [23]. In this view, quantifying the integrity of such brain systems (e.g. via neuroimaging, neurophysiology or circadian biology) along with their behavioural concomitants (e.g. neuropsychology, social cognition or sleep-wake patterns) may lead to the identification of objective markers of early risk states and also serve as treatment targets. For example, in a longitudinal study by our group, neuropsychological performance at baseline was the single best predictor of socio-occupational functioning at follow up, over and above diagnosis and symptom severity [24]. Such findings demonstrate the relevance of objective ‘brain’ markers (in this case, a cognitive phenotype) to provide important insights about a crucial functional domain, which cut across diagnostic categories to direct effective treatment strategies at the pathophysiological driver of poor patient outcomes.

Here, we present a systematic review of the neurobiological and neurocognitive correlates, of five functional domains in young people with mood and anxiety disorders. We focus specifically on major depression, bipolar disorder (I, II, not otherwise specified; NOS) and anxiety disorders (excluding post-traumatic stress disorder), since these most closely relate to the common developmental trajectories of emerging mood disorders in young people [25]. In this review we evaluate the relationship between the functional domains (described above), and evidence from neuropsychology, neuroimaging, sleep-wake and circadian biology, neurophysiology and metabolic studies. A wide age range was chosen (12–30 years) to focus on the adolescent and young adult population; referred to collectively as ‘young people’, to better understand the primary age group that are vulnerable and present to primary youth mental health services. The primary objective of this study is to establish the current status of the literature of young people with mood and anxiety disorder with respect to neurobiological investigations addressing any of the proposed five functional domains. Whilst, we expect that the large majority of identified studies would investigate clinical syndromes and a smaller number would investigate the remaining functional domains, it is expected that unique associations between neurobiological parameters and a functional domain, not accounted for by the clinical syndrome, will become clearer. The aim of our approach is to ultimately provide a framework for guiding the development of personalised assessment and interventions to prevent or delay significant disability in young mental health patients.

## Methods

Methods of review regarding eligibility criteria, data collection and synthesis were specified in advance in the form of a review protocol. We followed the guidelines for conducting and reporting a systematic review set out by ‘the PRISMA statement’ [26], and the ‘Cochrane Handbook for Systematic Reviews’ [27].

### Eligibility criteria

#### Report characteristics and information sources

We searched PubMed databases for unique records using the following criteria: (i) published in the last 20 years (i.e. between January 1994 and March, 2014, to coincide with the release of DSM-IV since this version introduced the use of clinical significance ratings related to the impact of illness on areas of functioning); (ii) the study was reported in English; and (iii) had keyword combinations (see Table 1 for full search terms). The reference lists of studies identified by our PubMed search were not utilised as an additional information source.

**Table 1:** Full list of search terms used according to each topic area

	Topic area	Pub Med Terms
*Population of interest*	Mood and anxiety syndrome or profile	Anxiety disorder OR anxiety OR depression OR depressive disorders OR depressive disorder [MeSH Terms] OR major depressive disorder OR MDD OR disorder, bipolar [MeSH Terms] OR bipolar disorder OR affective disorder OR mood disorder OR affective syndrome OR manic syndrome OR depressive syndrome OR anxious syndrome
	Youth	Adolescents OR young people OR adolescence OR adolescent [MeSH Terms] OR youth OR young adult
*Functional domain*	Social and economic participation	Socio-occupational functioning OR functioning OR social functioning OR occupational participation OR economic participation
	Physical health	Physical health OR metabolic rate OR obesity OR blood pressure OR CVD OR fitness OR cardiovascular disease OR BMI or body mass index OR waist measurement OR blood glucose OR smoking rate OR physical activity OR cholesterol levels
	Suicide and self-harm behaviours	Suicide [MeSH Terms] OR suicide ideation OR self-harm OR suicide risk
	Alcohol and substance use	Substance use disorder [MeSH Terms] OR alcohol use OR drug use
	Clinical syndrome	Illness progression OR syndrome progression OR symptom severity
*Neurobiological parameter*	Neuropsychology	Neuropsychology OR neuropsychological test [MeSH Terms]
	Imaging	Brain imaging [MeSH Terms] OR imaging OR neuroimaging OR fMRI OR DTI OR MRI OR MRS
	Sleep-wake and circadian biology	Actigraphy [MeSH Terms] OR melatonin secretion OR circadian rhythms OR DLMO OR sleep-wake and circadian biology
	Neurophysiology	Neurophysiology OR EEG OR electroencephalography OR ERP OR event-related potentials
	Metabolic	BMI OR waist measurement OR blood pressure OR cholesterol

*Note.* Terms within each cell in column 3 (above) used the ‘OR’ function, whilst the ‘AND’ function was used to combine terms between the cells of column 3

#### Study characteristics and selection

Using a pro forma, the first author (FI) checked the abstract and/or full texts of each paper for the following inclusion criteria; i) a mean age between 12 and 30 years; ii) at least one group of subjects was reported as having a primary mood and anxiety (i.e. depression, bipolar, anxiety) disorder (according to DSM-IV or ICD-10 criteria) or syndrome (e.g. ‘at risk’, current depressive symptoms); iii) at least one of the following functional domains: (a) social and economic participation; (b) physical health; (c) suicide and self-harm behaviours; (d) alcohol and substance use; and/or (e) clinical syndrome, was measured/quantified; and iv) a statistical (i.e. correlational, regression, etc.) association between the functional domain and at least one neurobiological parameter (i.e. neuropsychology, brain imaging, sleep-wake and circadian biology, neurophysiology and metabolic) was reported. Review articles and case studies were excluded from the final synthesis. Studies were labelled ‘yes’ if they fulfilled all four criteria, ‘no’ if they failed to meet all four criteria or ‘possibly’ if it was unclear whether all criteria were fully met. Any disagreement with these rules was resolved by consensus with the senior author (DH).

### Identification of studies

**Fig. 1:**
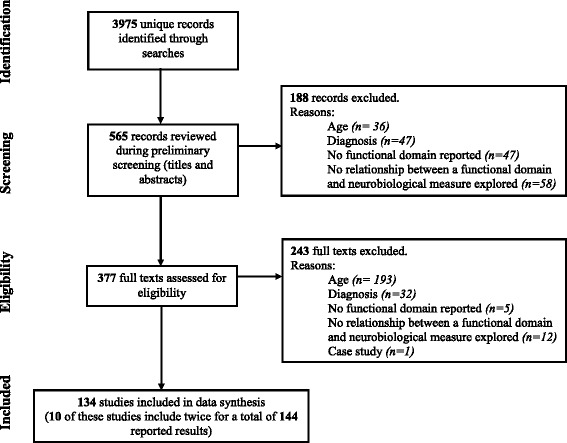
This figure communicates the flow of studies through the systematic review process and identifies the number of studies excluded at each phase as well as the reason for exclusion

Figure 1 displays the series of steps undertaken as we identified studies for this systematic review. First, of the 3975 studies identified by the searches (see Table 1 for search terms), 565 titles and abstracts were examined for eligibility. At this stage, 188 studies were excluded on the basis of not meeting one or more of the eligibility criteria specified. The eligibility stage involved the assessment 377 full texts (i.e. the published manuscript) to evaluate whether these studies were suitable, which led to a further 243 studies being excluded. The remaining 134 studies were included in the final synthesis (see Table 2 for a summary of these studies).

**Table 2:** Overview of the included studies organised according to functional domain and neurobiological parameter investigated

	*Neurobiological parameter*				
*Functional domain*	Neuropsychology	Imaging	Sleep-wake and circadian biology	Neurophysiology	Metabolic
Social & economic participation	Beiderman (2011)	Perlman (2012)	**Abelson (1996)**	***Kaur (2013)***	Taylor (2008)*
	Fujii (2013)*		Goodyer (1998)*		
	Korhonen (2002)		Granger (1994)		
	***Lee (2013c)***				
	***Lee (2013a)***				
Physical health		Bond (2011)		Jarworska (2011)*	Mannie (2013)
Suicide & self-harm behaviours	Bridge (2012)	Ehrlich (2004)	Coplan (2000)*	**Ashton (1994)**	Apter (1999)
	**Miranda (2012)**	Ehrlich (2005)	**Mathew (2003)**	Graae (1996)	De Berardis (2013)
	Ohmann (2008)	Goodman (2011)*	McCracken (1997)*	Pechtel (2013)	Plana (2010)
	Oldershaw (2009)	Pan (2011)			Soreni (1999)
	Pan (2013b)*	Pan (2013a)			Tyano (2006)
		Pan (2013b)*			
Alcohol & substance use	Harvey (2007)	*Chitty (2013)*		*Chitty (2014)*	Goldstein (2008)
	*Hermens (2013a)*	**Cornelius (2010)**		Ehlers (2011)	
		De Bellis (2005)			
		**Jarvis (2008)**			
		Medina (2007)			
Clinical syndrome	Andres (2007)	Adler (2007)	Adam (2010)	**Bakker (2011)**	Pine (2001)
	Andres (2008)	Aghajani (2013)	Ankers (2009)	Carrasco (2013a)	Taylor (2008)*
	Basso (2001)	Bitter (2011)	Armitage (1997)	Carrasco (2013b)	
	Cataldo (2005)	Chang (2008)	Coplan (2000)*	**Croarkin (2014)**	
	Fleck (2008)	Chu (2013)	Doane (2013)	Dai (2012)	
	Fujii (2013)*	Diler (2013)	Ellenbogen (2006)	El Badri (2001)	
	Gunther (2004)	Forbes (2006)	Ellenbogen (2010)	Hajcak (2008)	
	Han (2012)	Forbes (2010)	Goodyer (1998)*	Houston (2003)	
	Kıvırcık (2003)*	Gabbay (2012)	**Harkness (2011)**	Jarworska (2011)*	
	Klimkeit (2011)	Gabbay (2013)	Landsness (2011)	Jarworska (2013)	
	Okasha (2000)*	Gao (2013)	McCracken (1997)*	Kıvırcık (2003)*	
	Pavuluri (2010a)	**Gilbert (2000)**	Murray (2012)	Okasha (2000)*	
	**Schmid (2013)**	Gilbert (2009)	**Rao (1996)**	Stern (2010)	
	**Simons (2009)**	Goodman (2011)*	Rao (2008)	Vaidyanathan (2014)	
	Torres (2010)	Gruner (2012)	*Robillard (2013a)*		
	Wall (2013)	*Hatton (2012)*	*Robillard (2013b)*		
		Henderson (2013)	*Scott (2014)*		
		Ho (2014)			
		**Huang (2012)**			
		**Huyser (2011)**			
		Huyser (2013)			
		Ladouceur (2011)			
		*Lagopoulos (2012)*			
		*Lagopoulos (2013a)*			
		*Lagopoulos (2013b)*			
		**Lazaro (2008)**			
		**Lazaro (2012)**			
		**Lisy (2011)**			
		MacMaster (2006)			
		MacMillian (2003)			
		McClure (2007)			
		Meng (2013)			
		Pannekoek (2014)			
		Patel (2008)			
		Pavuluri (2010b)			
		Pavuluri (2011)			
		Phan (2013)			
		Rauch (2002)			
		Reynolds (2014)			
		Rosenberg (1997)			
		**Rosenberg (2000)**			
		Rosso (2005)			
		Schienle (2011)			
		Schneider (2012)			
		Strawn (2012)			
		Wegbreit (2011)			
		Yucel (2008)			
		Zarei (2011)			
		Zuo (2013)			

Note. * = indicates the study appears more than once, BOLD = longitudinal study, *italicized* = Study conducted by the Brain and Mind Centre

### Synthesis of results

For each of the included studies, the reviewer (FI) collated data with respect to the study design (i.e. cross-sectional, longitudinal; see Table 2), sample characteristics (i.e. age, sample sizes), aims, key measures (e.g. neuropsychological, circadian, clinical) and key findings (presented in Tables 3, 4, 5, 6 and 7; one table per neurobiological parameter). To clarify, the key findings for each study were taken as any evidence of an association between a particular neurobiological measure and a functional domain. In order to achieve this, the various scales, tests, and assessments were collapsed into broader categories of key measures (e.g. specific neuropsychological subtests grouped into a cognitive domain; see Tables 3, 4, 5, 6 and 7). Given the variability in methodology and the large and varied outcomes of identified studies it was not appropriate to carry out a meta-analysis [28].

**Table 3:** Neuropsychological studies evaluating the five functional domains in young people (12–30 yrs) with a mood and/or anxiety disorder

Outcome measure	Study	Age (mean ± SD)	Sample (N)	Aims	Key measures	Key findings
*Social and economic participation*	[40]	HC: 13.6 ± 2.1,	HC (47M; 34F),	Evaluate the clinical impact of executive function deficits in youth with BPD-I disorder.	NΨ: Executive function deficits (CPT, CVLT-C, RCF, SCWT, WCST, WAIS-III- FFD)	BPD-I: ↓ executive function ~ ↓ social and economic participation
		HC-EFD: 13.9 ± 2.3,	HC-EFD (12M; 5F),			
		BPD-I: 13.7 ± 2.1,	BPD-I (52M; 24F),			
		BPD-I-EFD: 12.8 ± 2.4	BPD-I-EFD (49M; 13F)		Functional: GAF, WRAT-III, placement in special class	
	[39]*	SAD: 23.9 ± 6.7;	SAD (20M; 10F)	Assess the neuropsychological function of SAD without co-morbidity	NΨ: Executive function (CPT, TMT-B, WCST), Processing speed (TMT-A), Verbal learning & memory (AVLT)	SAD: ↓ executive function ~ ↓ social and economic participation (and ↑ SAD severity)
		HC: 25.6 ± 5.6	HC (20M; 10F)			
					Functional: GAF	
	[29]	MDD: 18.9 ± 2.0,	MDD (4M; 12F),	Investigate the association between cognitive performance and MDD.	NΨ: Executive function (SCWT, TMT-B), Verbal learning & memory (WMS-SR, LLT, RCF-3min), General intellect (WAIS-III-S & V), Attention (WAIS-III- DS, BD & DSp)	MDD: No significant ~ NΨ
		HC: 16.9 ± 1.9	HC (11M; 14F)			MDD: ↓ social and economic participation
					Functional: GAF	
		FED: 22.00 ± 4.9	FED (8M; 12F)	Assess the effectiveness of CR in patients with a first-episode of either major depression or psychosis	NΨ: Executive function (CANTAB-IED; -FAS, TMT-B), Processing speed (TMT-A, category fluency), Attention and working memory (LDSF, LDSB, CANTAB-SSP;-RVP, mental control), Immediate learning and memory (Logical Memory I, RAVLT- tot, CANTAB-PAL), Delayed learning and memory (LM-Ret, RCF-3min, RAVLT-Ret)	FED & FEP: CR ~ ↑ immediate learning and memory, and ↑ social and economic participation (mediated by ↑ delayed learning and memory)
		FEP: 23.30 ± 3.9	FEP (20M; 13F)			
					Functional: SFS	
	[24]	MHP: 21.6 ± 4.5	MDD (34)	Identify cognitive markers that predict later socio-occupational functioning.	NΨ : Executive function (CANTAB-IED, TMT-B), Processing speed (TMT-A, CANTAB-FAS), Attention and working memory (CANTAB-RVP), Verbal learning & memory (LM-Ret, RAVLT-ret), Visual learning & memory (CANTAB- SSP;- PAL)	MHP: ↑ BL general NΨ ~ ↑ social and economic participation at FUP
			BPD (29)			
			PSD (30)			
			(of the total 93, 52 % were male)			
					Functional: SOFAS	
*Suicide and self-harm*	[31]	SA: 15.5 ± 1.4	SA (10M; 30F)	Examine decision-making processes in suicide attempters and never-suicidal comparison subjects	NΨ: Decision making (IGT)	SA: ↓ decision making ~ suicide attempt history
		PC: 15.6 ± 1.4	PC (10M; 30F)		Functional: CSHF, PSIS	
	[32]	SA: 18.31 ± 0.63	SA (1M; 12F)	Examine whether cognitive inflexibility can differentially and prospectively predict suicidal ideation.	NΨ: Executive function (WCST; perseverative errors)	SA: ↓ BL cognitive inflexibility ~ ↑ suicide ideation at 6-month FUP.
		NSA: 18.31 ± 0.78	NSA (9M; 23F)			
					Functional: BSS, SBS, SHBQ	
	[35]	SIB: 15.5 ± 1.3	SIB (99)	Investigate the neuropsychological differences between psychiatric patients with and without SIB.	NΨ: Executive function (SCWT, WCST)	Null findings
		NSIB: 15.1 ± 1.4	NSIB (77)			
					Functional: Clinical interview	
	[33]	HC: 15.8 ± 1.5	HC (11M; 46F)	Assess decision making and problem solving ability in adolescents with current or past self-harm	NΨ: Decision making (IGT, MEPS)	DSH: ↓ decision making ~ current, but not past DSH
		PC: 15.7 + 1.3	PC (2M; 20F)		Functional: Clinical interview	
		DSH: 15.8 + 1.5	DSH (5M; 49F)			
	[34]*	SA: 16.20 ± 0.78	SA (4M; 11F)	Measure neural activity during performance on the IGT in adolescents.	NΨ: Decision making (IGT-mod)	SA: ↑ decision making ~ suicide attempt history
		PC: 15.79 ± 1.58	PC (7M; 7F)		Functional: C-CASA, CSHF, SIQ, SIS	
		HC: 15.15 ± 1.46	HC (8M; 5F)			
*Alcohol and substance use*	[164]	CU: 16.2 (13.5 – 18.4)	CU (28M; 42F)	Investigate the non-acute relationship between cannabis use and cognitive function	NΨ: Intelligence (WASI), Executive function (CANTAB-IED), (CANTAB-MS), Attention and working memory (CANTAB-RVP;-SWM; -SSP, DS, SDMT), Immediate learning and memory (RAVLT, CANTAB-PAL)	CU: ↓ attention, spatial working memory and learning.
						CU: was independent predictor of performance on the working memory and strategy measures
					Functional: TLFB	
	[165]	HC-NB: 22.9 ± 3.1	HC-NB (7M; 14F)	Compare the cognition in binge drinkers with depression to those with depression alone or binge drinking alone.	NΨ: Intelligence (WTAR), Psychomotor speed (TMT-A) Executive function (TMT-B), Verbal learning and memory (RAVLT), Attention (CANTAB- RVP), working memory (CANTAB-SSP, Visuospatial learning and memory (CANTAB-PAL)	MDD-B: ↓ visual learning & memory and overall pattern of ↓ NΨ functioning.
		HC-B: 23.0 ± 2.5;	HC-B (13M; 11F)			
		MDD-NB: 21.7 ± 3.2	MDD-NB (16M; 32F)			
		MDD-B: 21.8 ± 3.4	MDD-B (24M; 19F)			
					Functional: AUDIT	
*Clinical syndrome*	[50]	OCD: 13.84 ± 2.78	OCD (18M; 17F)	Investigate the influence of clinical variables treatment on cognitive performance in OCD patients	NΨ: Intelligence (WISC-R: Vo), Visual organisation (WISC-R:-BD), Attention (WISC-R: -DS;-Co), Verbal learning and memory (WMS-III- LM1 & 2, RAVLT), Visual learning and memory (WMS-III: VR 1 & 2, RCFT), Processing speed (TMT-A), Cognitive flexibility (TMT-B, WCST, SCWT), Verbal fluency (COWAT)	OCD: ↓ verbal and visual memory and velocity. (Neuropsychological impairment was not related to obsessive-compulsive severity)
		HC: 13.81 ± 2.74	HC (18M; 17F)			
					Clinical: CDI, Y-BOCS	
	[51]	OCD: 13.46 ± 2.83	OCD (16M; 13F)	Explore the evolution of cognitive dysfunction in children and adolescents with OCD after treatment	NΨ: Intelligence (WISC-R: Vo), Visual organisation (WISC-R:-BD), Attention (WISC-R: -DS;-Co), Verbal learning and memory (WMS-III- LM1 & 2, RAVLT), Visual learning and memory (WMS-III: VR 1 & 2, RCFT), Processing speed (TMT-A), Cognitive flexibility (TMT-B, WCST, SCWT), Verbal fluency (COWAT)	OCD: ↓ memory, speed of information processing and cognitive flexibility. (After treatment the cognitive profile of the OCD group was normalized)
		HC: 13.06 ± 2.84	HC (12M; 10F)			
					Clinical: Y-BOCS	
	[53]	OCD: 29.70 ± 10.74	OCD (12M; 8F)	Examine the impact of depression on executive function deficits in OCD	NΨ: VCAT, Verbal Fluency (COWAT), Processing speed (TMT-A), Cognitive flexibility (TMT-B, WCST)	OCD: cognitive flexibility deficits ~ co-morbid depression severity
		HC: 30.06 ± 10.06	HC (11M; 21F)			
					Clinical: MMPI-D	
	[47]	HC: 12.5 ± 2.4	HC (11M; 10F)	Compare impulsivity at the neuropsychological and behavioural level in young depressed patients and healthy controls.	NΨ: Cognitive style (MFFT), Verbal fluency (VFT), Decision making (WDWT), cognitive flexibility (SCWT), Impulsivity (CPT)	DD: ↑ symptom severity ~ ↑ reaction time, ↓ in commission errors.
		DD: 11.7 ± 2.3	DD (11M; 10)			
						DD: ↑ conservative response styles & attention problems, ↓ reaction times & response initiation
					Clinical: HDRS, CDI, CPRS-R:L	
	[166]	HC: 28.2 ± 7.9	HC (20M; 28F)	Investigate the effect of syndrome state or course on executive dysfunction	NΨ: Intelligence (NAART), Cognitive flexibility (WCST)	EUT: ↑ cognitive flexibility than MEM. Performed similarly to FEM
		EUT: 30.0 ± 7.2	EUT (11M; 14F)			
		FEM: 25.7 ± 9.2	FEM (11M; 10F)		Clinical: YMRS, HDRS	
		MEM: 28.2 8.6	MEM (16M; 18F)			
	[39]*	SAD: 23.9 ± 6.7;	SAD (20M; 10F)	Assess the neuropsychological function of SAD without co-morbidity	NΨ: Executive function (CPT, TMT-B, WCST), Processing speed (TMT-A), Verbal learning & memory (AVLT)	SAD: ↓ executive function ~ ↑ SAD severity
		HC: 25.6 ± 5.6	HC (20M; 10F)			
					Clinical: GAF	
	[44]	HC: 12.8 ± 2.5	HC (15M; 18F)	Examine basic performance neuropsychological performance in children and adolescents with anxiety disorder or depressive disorder and in healthy subjects under drug-free condition	NΨ: Intelligence (WISC-III), Verbal learning and memory (RAVLT), Attention (go-no go task)	DD: ↓ verbal learning and memory compared to HC and ANX.
		ANX: 12.4 ± 2.3	ANX (19M; 15F)			
		DD: 13.5 ± 2.6	DD (17M; 14F)			
					Clinical: CDI	
	[48]	HC: 17.46 ± 1.59, MDD: 17.32 ± 1.59	HC (14M, 16F) MDD (12M, 19F)	Investigate whether major depression in adolescence is characterized by neurocognitive deficits in attention, affective decision making, and cognitive control of emotion processing	NΨ: Inhibitory control (CPT, go-no go task), Attention (ANT), Decision making (IGT), Verbal learning and memory (RAVLT), Attention (go-no go task)	MDD: ↑ depression symptom severity ~ ↓ cognitive control of emotion processing
					Clinical: BDI	
	[167]*	OCD: 27 ± 9.8	OCD (15M; 16F)	Characterize the cognitive functions of the patients with OCD by utilizing ERPs and neuropsychological tests	NΨ: cognitive flexibility (SCWT, TMT-B), Processing speed (TMT-A), Design fluency test, Verbal fluency (CWAT)	Null findings for neuropsychological tests.
		HC: 27.4 ± 9.1	HC (14M; 16F)			
					Clinical: HDRS	
	[49]	MDD: 15.3 ± 1.6	MDD (5M; 17F)	Investigate verbal fluency, cognitive speed, motor speed, and executive functions in adolescents with unipolar depression.	NΨ: Verbal fluency (COWAT), Processing speed (Inspection time task), Working memory (Serial choice reaction time task), Set shifting (Local-global task) Clinical:	DD: ↓ WM & VF. MDD: ↓WM & processing speed
		DD: 15.6 ± 1.5	DD (6M; 6F)			
		HC: 15.8 ± 1.2	HC (9M; 24F)			
	[52]	OCD: 24.06 ± 5	OCD (21M; 9F)	Assess the relationship between cognitive dysfunction, clinical status and severity in OCD.	NΨ: Intelligence (WAIS-BD; -S), Cognitive flexibility (WCST)	Results showed a defective visuospatial recognition, which worsens with chronicity, deteriorated set-shifting abilities, overfocused attention to irrelevant stimuli and delayed selective attention to relevant tasks. Mild cases showed better selective attention than severe cases. Obsessive cases had a defective visual memory, while compulsive cases had delayed perception of task relevant stimuli. Mixed cases showed disturbed information-processing both early and late.
		HC: Matched	HC (21M; 9F)			
					Clinical: YBOCS	
	[55]	HC: 12.4 ± 3.3,	HC (15M; 9F)	Examine the treatment impact of lamotrigine on the neurocognitive profile of patients with pediatric bipolar disorder	NΨ: Attention (TMT-A, CPT), cognitive flexibility (TMT-B), Verbal fluency (COWT), Working memory (WMS; DS, SS), Verbal memory (CVLT)	BPD: ↑ Working memory and verbal memory following treatment (to levels similar to HC)
		BPD: 13 ± 3.1	BPD (18M; 16F)			
					Clinical: YMRS	
	[168]	MDD: 26.93 ± 5.33	MDD (14M; 14F)	Assess the association between executive function and relapse	NΨ: cognitive flexibility (CWIT, TMT-B), Verbal fluency (VFT), Processing speed (TMT-A),	MDD: Poor BL inhibition and switching ~ ↑ relapse at FU
		HC: 26.93 ± 5.18	HC (14M; 14F)			
					Clinical: MADRS	
	[45]	CS: 28 ± 7.9	CS (642)	Examine whether cognitive deficits predict current and/or follow-up (sub)clinical depressive symptoms in the general population	NΨ: Information processing (SCWT, CST, LDST), Episodic memory (AVLT)	CS: Poor BL episodic memory ~ ↑ depressive symptoms at FUP
					Clinical: SCL-90	
	[169]	BD: 22.2 ± 3.9	BD (23M; 22F)	Determine whether neuropsychological impairments are present in clinically stable patients with bipolar disorder shortly after resolution of their first manic episode	NΨ: Intelligence (NAART), Visual spatial reasoning (K-BIT), Attention/processing speed (TMT-A, CANTAB-RVP, CVLT), Learning and memory (CVLT- recall, CANTAB-SRM;-PRM;-PAL), cognitive flexibility (TMT-B, CANTAB-IED;-SWM), Verbal fluency (COWT),	BPD: ↓ learning/memory, spatial/nonverbal reasoning, executive function, and some aspects of attention
		HC: 22.5 ± 4.8	HC (12M; 13F)			
					Clinical: PANSS, HDRS, BPRS, GAF, YMRS	
	[56]	MDD: 16.2 ± 1.1	MDD (7M; 11F)	Investigate the neurocognitive outcome in adolescents who were treated with TMS	NΨ: Verbal learning and memory (CAVLT), Cognitive flexibility (D-KEFS, TMT)	MDD: ↓ Depressive symptoms were associated with ↑ in immediate and delayed verbal memory.
					Clinical: CDRS-R	

Note. *Sample*: *ANX* Anxiety disorder, *BPD* Bipolar Disorder, *BPD-I* Bipolar Disorder I, *BPD-I-EFD* Bipolar Disorder I with Executive Function Deficits, *CS* Community Sample, *DD* Depressive disorder, *CU* Cannabis user, *DSH* Deliberate Self-Harm, *EUT* euthymic, *FED* First-Episode depression, *FEM* first episode mania, *FEP* First-Episode Psychosis, *HC* Healthy Controls, *HC-B* Healthy Control Binge drinker, *HC-EFD* Healthy Control with Executive Function Deficits, *HC-NB* Healthy Control Non Binge drinker, *MDD* Major Depression Disorder, *MDD-B* Major Depression Disorder Binge drinker, *MDD-NB* Major Depression Disorder Non Binge drinker, *MEM* multiple episode mania, *MHP* Mental Health Patients (mixed diagnosis sample), *NSA* No Suicide Attempt, *NSIB* No Suicide Ideation Behaviour, *OCD* Obsessive Compulsive Disorder, *PC* Psychiatric Control (i.e. psychiatric diagnosis but no suicide attempt), *PSD* Psychotic Spectrum Disorder, *SA* Suicide Attempters, *SAD* Social Anxiety Disorder, *SIB* Suicide Ideation Behaviour

*Measures*: *ANT* Attention network test, *AUDIT* Alcohol Use Disorder Identification Test, *AVLT* Auditory Verbal Learning Test, *BDI* Beck Depression Inventory, *BPRS* Brief psychiatric rating scale, *BSS* Beck Scale for Suicidal Ideation, *CANTAB* Cambridge Neuropsychological Test Automated Battery [subsets include: *FAS* Fluency and semantic test, *IED* Intra/Extra dimensional Set Shift Errors, *MS* Motor Screening, *PAL* Paired associates learning, *PRM* Pattern recognition memory, *RVP* Rapid Visual Processing hits score, *SRM* Spatial recognition memory, *SSP* Spatial span task, *SWM* Spatial working memory), *C-CASA* Columbia Classification Algorithm of Suicide Assessment, *CDI* Children’s Depression Inventory, *COWAT* Controlled Oral Word Association Task, *CPRS-R:L* Conners Parent Rating Scale- Revised: Long Version, *CPT* Continuous Performance Test, *CSHF* Colombia Suicide History Form, *CST* Concept shifting test, *CVLT* California Verbal Learning Test for Children, *D-KEFS* Delis–Kaplan Executive Function System, *GAF* Global Assessment of Functioning, *HDRS* Hamilton depression rating scale, *IGT* Iowa Gambling Task, *K-BIT* Kaufman Brief Intelligence Test, *LDSB* Longest Digit Span Backward, *LDSF* Longest Digit Span Forward, *LDST* letter digit substitution test, *LM-Ret* logical memory- percentage retention, *MADRS* Montgomery-Asberg Depression Rating Scale, *MEPS* means-ends problem-solving procedure, *MFFT* matching familiar figures test, *MMPI-D* Minnesota Multiphasic Personality Test, depression subscale, *NAART* North American Adult Reading Test, *NΨ* neuropsychological, *PANSS* positive and negative syndrome scale, *PSIS* pierce suicide intent scale, *RAVLT* rey auditory verbal learning test; total score; retention; and/or 20min score, *RCF* Rey-Osterrieth Complex Figure, *SBS* Suicide Behavior Screening, *SCWT* stroop colour and word test, *SCL-90* symptom checklist, *SCWT* stroop colour and word test, *SDMT* symbol digit modality test, *SFS* social functioning scale, *SHBQ* self-harm behavior questionnaire, *SIQ* suicide ideation questionnaire, *SIS* suicide intent scale, *SOFAS* social and occupational functioning assessment scale, = time-line followback, *TMT-A* trail making test – part A, *TMT-B* trail making test – part B, *VFT* verbal fluency task, *WAIS-III* Wechsler Adult Intelligence Scale [subsets include: *S* similarities, *V* Vocabulary, *DS* Digit symbol, *BD* Block design, *FFD* freedom from distractibility], *WASI* Wechsler abbreviated scale of Intelligence, *WISC* Wechsler Intelligence Scale For Children, *WCST* Wisconsin Card Sorting Test, *WDWT* Walk don’t-walk test, *WMS* Wechsler Memory Scale [subsets include: *DS* Digit Span, *LM* logical memory 1 & 2, SR = story recall, *LLT* List Learning Test, *SS* Spatial span, *VR* Visual Reproduction], *WRAT-III* wide range achievement test – third edition, *WTAR* wechsler test of adult reading, *Y-BOCS* Yale–Brown obsessive-compulsive scale, *YMRS* young mania rating scale

*Findings:* ↑ = Increased, Improved or Higher, ↓ = Decreased, Reduced or Lower, ~ = ‘is associated with’, *FUP* follow-up

*indicates that the study features more than once in the data synthesis

**Table 4:** Imaging studies evaluating the five functional domains young people (12-30 yrs) with a mood and/or anxiety disorder

Outcome measure	Study	Age (mean ± SD)	Sample (N)	Aims	Key measures	Key findings
*Social and economic participation*	[57]	MDD: 15.7 ± 1.5;	MDD (8M; 6F)	Assess amygdala activation and connectivity during an emotional regulation task.	Imaging: fMRI	MDD: ↓ amygdala–seeded connectivities ~ ↓ social and economic participation
		HC: 15.1 ± 1.6	HC (8M; 6F)		Functional: CGAS	
*Physical health*	[58]	BPD-O: 23.8 ± 4.5	BPD-O (9M; 11F)	Examine the relationship between BMI and brain volumes in mania.	Imaging: sMRI	BPD: ↑ BMI ~ ↓ WMV and TLV
		BPD-N: 22.2 ± 4.4	BPD-N (19M; 18F)		Functional: BMI	HC: ↑ BMI ~ ↓ TBV and GMV.
		HC-O: 22.0 ± 3.8	HC-O (12M; 5F)			
		HC-N: 22.3 ± 3.5	HC-N (19M; 19F)			
*Suicide and self-harm*	[170]	MHP: 14.6 ± 3.4	PSD (18M; 5F)	Compare WMH in psychiatrically hospitalized youth with and without a history of suicide attempt	Imaging: sMRI	MDD: ↑ WMH ~ suicide attempt history, but not ideation
			BPD (26M; 9F) MDD (33M; 15F)		Functional: PRS	
			PC (34M; 12F)			
	[171]	MDD: 26.7 ± 5.5	MDD (34M; 68F)	Compare the prevalence and location of WMH in young MDD inpatients with and without histories of suicide attempts	Imaging: sMRI	MDD: ↑ PVH, not DWMH, ~ suicide attempt history, but not ideation
					Functional: Clinical records	
	[59]*	HC: 16.2 ± 0.8	HC (4M; 9F)	Evaluate the ACC volumes of MDD/borderline personality patients with and without a suicide attempt history	Imaging: sMRI	MDDx: ↓ BA24 volumes ~ ↑ number of suicide attempts (and ↑ borderline severity, but not depression)
		MDDx: 15.8 ± 1.1	MDDx (2M; 11F)		Functional: Clinical interview	
	[62]	SA: 16.20 ± 0.78 PC: 15.87 ± 1.55 HC: 15.21 ± 1.42	SA (4M; 11F)	Evaluate the association between neural activity during performance of the go no-go task and suicide history.	Imaging: fMRI	PC: ↑ activity in right ACG compared to SA (but SA not different from HC)
			PC (7M; 8F)		Functional: CSHF	
			HC (8M; 6F)			
	[60]	SA: 16.21 ± 0.80	SA (4M; 10)	Measure neural activity during processing of emotional faces in adolescents with a history of depression and suicide attempt	Imaging: fMRI	SA: ↑ dorsal ACG activity when viewing angry faces, and ↓ visual, sensory, prefrontal, ACG activity to intense happy and neutral faces ~ suicide attempt history.
		PC: 15.87 ± 1.55	PC (7M; 8F)		Clinical: C-CASA, CSHF, SIQ, SIS	
		HC: 15.27 ± 1.39	HC (8M; 7F)			
	[34]*	SA: 16.20 ± 0.78	SA (4M; 11F)	Measure neural activity during performance on the IGT in adolescents.	Imaging: fMRI	PC: ↑ hippocampal activity compared to HC. (HC and SA did not differ, evidence of ↓ activation)
		PC: 15.79 ± 1.58	PC (7M; 7F)		Functional: C-CASA, CSHF, SIQ, SIS	
		HC: 15.15 ± 1.46	HC (8M; 5F)			
*Alcohol and substance use*	[172]	BPD-L: 23.7 ± 3.6	BPD-L (14M; 5F)	Assess the effects of alcohol use on GSH in young people with BPD.	Imaging: MRS	BPD-H: ↓ GSH
		BPD-H: 23.4 ± 3.1	BPD-H (12M; 2F)		Functional: AUDIT	
		HC: 23.6 ± 2.8	HC (13M; 4F)			
	[65]	MDD: 21.7 ± 2.0	MDD (5M; 1F)	Examine the effect of cannabis use on threat-related amygdala reactivity.	Imaging: fMRI	MDD: ↑ CU ~ ↓amygdala reactivity
					Functional: SCID (presence of dependence)	
	[66]	AUD: 17 ± 2.1	AUD (8M; 6F)	Compare prefrontal-thalamic-cerebellar measures of adolescents and young adults with adolescent-onset alcohol use disorders	Imaging: sMRI	AUD: ↓ PFC & PFC WMV.
		HC: 16.9 ± 2.3	HC (16M; 12F)		Fucntional: ACQ, LHAUI, SCID	AUD: ↓ PFC GM ~ ↑ alcohol consumption
						AUD(M): ↓ CV
						PFC volume variables ~ measures of alcohol consumption
	[64]	BPD: 16 ± 2,	BPD (5M; 9F)	Compare brain morphometry in bipolar adolescents with co-occurring substance and alcohol disorders	Imaging: sMRI	BPD: CUD ~ ↓ LFG GMV & ↑ RC, PCG GMV
					Functional: ASI, SCID, SAC	
	[63]	CU: 18 ± 0.7	CU (12M; 4F)	Examine the relationship between brain volumes, depression and cannabis use.	Imaging: sMRI	CU: ↓ WMV ~ ↑ depressive symptoms
		HC: 18 ± 0.9	HC (11M; 5F)		Functional: BDI, CDDR, HDRS, TLFB,	
*Clinical syndrome*	[173]	BPD: 19.9 ± 7.9	BPD (15M; 18F)	Explore structural brain changes in first-episode bipolar patients	Imaging: VBM	BPD: ↑ volume in left thalamus and fusiform and cerebellum bilaterally. ↑ AC, PPS GMD. ↑ middle/superior temporal and posterior cingulate gyri, GMV & GMD.
		HC: 21.5 ± 4.3	HC (19M; 14F)		Clinical: SCID, KSADS	
	[90]	DD: 15.6 ± 1.4,	DD (4M; 21F)	Investigate WM microstructure in a sample of clinically depressed adolescents relative to matched controls.	Imaging: DTI	DD: ↓ FA and ↑ RD, MD in corpus callosum. ↑ FA & AD, and ↓ RD in uncinate fasciculus.
		HC: 14.7 ± 1.6	HC (3M; 18F)		Clinical: ADIS, CDI, CBCL, RCADS, YSRS	
	[46]	BPD: 15.8 ± 1.8	BPD (6M; 11F)	Compare amygdala neurodevelopment among BPD, ADHD, and healthy adolescents	Imaging: sMRI	BPD: ↑ BL amygdala volumes ~ symptomatic recover compared to those who did not achieve recovery. No increase in amygdala volume over time.
		ADHD: 16.3 ± 1.7	ADHD (13M; 11F)		Clinical: KSADS, LIFE, YMRS, HDRS	
		HC: 16.3 ± 1.8	HC (13M; 10F)			
	[85]	BPD: 15.9 ± 1.4	BPD (4M; 4F)	Evaluate the effect of lamotrigine treatment on amygdalar activation	Imaging: fMRI	BPD: clinical improvement ~ ↓ right amygdalar activation
					Clinical: CDRS	
	[99]	BPD: 25 ± 9	BPD (7M; 14F)	Investigate the distribution of lactate in bipolar and healthy brains	Imaging: MRS	BPD: ↑ Lac/NAA & Lac/Cr ratio
		HC: 25 ± 6	HC (5M, 5F)		Clinical: SCID	
	[174]	BPD: 15.6 ± 0.9	BPD (2M; 8F)	Explore the neural correlates of depression at baseline and after 6 weeks of open as usual treatment	Imaging: fMRI	BPD: After treatment, ↓ left occipital cortex activity in the intense fearful experiment, but ↑ left insula, left cerebellum, and right ventrolateral PFC in the intense happy experiment. ↑ improvement in depression ~ ↑ BL activity in ventral ACC to mild happy faces
		HC: 15.6 ± 1.2	HC (2M; 8F)		Clinical: KSADS, CDRS, SCARED, YMRS	
	[175]	MDD: 14.73 ± 1.49	MDD (3M; 11F)	Examine behavioral and neural responses to reward in young people with depressive disorders using a reward decision-making task	Imaging: fMRI	MDD: ↓ activation in the ACC, bilateral caudate, and inferior OFC bilaterally during reward decision/anticipation and reward outcome.
		HC: 14.45 ± 1.79	HC (7M; 10F)		Clinical: KSADS, CBCL, SCARED, CDI, BDI	
	[176]	MDD: 12.9 ± 2.3	MDD (4M; 9F)	Evaluate reward-related brain function as a predictor of treatment response in adolescents with MDD	Imaging: fMRI	MDD: severity, anxiety and depression symptoms ↓ following treatment. ↑ reward related striatal function before treatment ~ ↑ clinical severity, ↓ anxiety symptoms and faster improvement in anxiety symptoms after treatment. ↑ mPFC function before ~ slower improvements in anxiety symptoms.
					Clinical: KSADS, MFQ, SCARED, CGI	
	[68]	MDD: 16.7 ± 2.7	MDD (9M; 12F)	Test whether ACC GABA levels are decreased in adolescents with MDD	Imaging: MRS, sMRI	MDD & HC: ↑ ACC GABA ~ ↓ anhedonia scores.
		HC: 16.2 ± 1.6	HC (6M; 15F)		Clinical: KSADS, CDRS-R, BDI	
						MDD: ↓ ACC GABA, ↓ ACC WM
	[177]	MDD: 17.1 ± 2.5	MDD (9M; 12F)	Assess striatum-based circuitry in relation to categorical diagnosis of MDD and anhedonia severity	Imaging: fMRI	MDD: ↑ iFC between all striatal regions bilaterally and DmPFC, RVC and ACC. MDD severity ~ iFC between the striatum and the precuneus, posterior cingulate cortex and dmPFC. Anhedonia severity ~ Pregenual ACC, subgenual ACC, supplementary motor area, and supramarginal gyrus iFC.
		HC: 16.3 ± 1.4	HC (9M; 12F)		Clinical: KSADS, CDRS-R, BDI	
	[178]	BPD: 15.1 ± 1.81	BPD (6M; 12F)	Investigate the brain structural changes in BPD children and adolescents	Imaging: DTI	BPD: ↓ GMV in left hippocampus. ↓ FA value in rACC.
		HC: 14.1 ± 1.61	HC (6M; 12F)		Clinical: KSADS, YMRS, MFQ	
						↓ hippocampal volume ~ ↑ YMRS score
	[91]	OCD: 12.35 ± 2.93	OCD (7M; 14F)	Measure neuroanatomical changes in the thalamus of patients with OCD near the onset of illness, and before and after treatment.	Imaging: MRI	OCD: ↑ thalamic volumes in treatment naïve patients. ↓ thalamic volumes (to comparable levels with controls) ~ paroxetine monotherapy.
		HC: 12.47 ± 8.33	HC (7M; 14F)		Clinical: KSADS, YBOCS, HDRS	
						↓ thalamic volumes ~ ↓ OCD symptom severity
	[179]	OCD: 13.1 ± 2.5	OCD (11M; 7F)	Examine whether overlapping but symptom dimension-specific neural activity patterns in adults are apparent in youths	Imaging: fMRI	OCD: ↓ activity in right insula, putamen, thalamus, dorsolateral prefrontal cortex and left orbitofrontal cortex, and right thalamus and right insula. ↑ OCD symptom related measures were significantly predictive of ↓ neural activity in the right dorsolateral prefrontal cortex during the contamination experiment.
		HC: 13.6 ± 2.4	HC (11M; 7F)		Clinical: YBOCS	
	[59]*	HC: 16.2 ± 0.8	HC (4M; 9F)	Evaluate the ACC volumes of MDD/borderline personality patients with and without a suicide attempt history	Imaging: sMRI	MDDx: ↓ BA24 volumes ~ ↑ borderline severity, but not depression
		MDDx: 15.8 ± 1.1	MDDx (2M; 11F)		Clinical: Clinical interview	
	[94]	OCD: 14.3 ± 2.1	OCD (13M; 10F)	Investigate white matter abnormalities in pediatric obsessive-compulsive disorder.	Imaging: DTI	ODD: ↑ FA in splenium ~ ↑ obsession severity
		HC: 14.2 ± 2.2	HC (12M; 11F)		Clinical: YBOCS, KSADS	
	[180]	MHP: 22.3 ± 3.7	MHP (50M; 83F)	Examine the relationship between anterior insula GMV, clinical symptom severity and neuropsychological performance.	Imaging: sMRI	MHP: ↓ GMV in left anterior insula. Changes (↑ or ↓) in right anterior insula GMV ~ ↑ symptom severity.
		HC: 23.8 ± 2.4	HC (13M; 26F)		Clinical: BPRS, HDRS, SOFAS	
	[95]	MDD: 16.8 ± 2.2	MDD (9M; 8F)	Investigate WM microstructure in MDD using diffusion tensor imaging	Imaging: DTI	MDD: ↓ WM integrity in the genu of corpus callosum, anterior thalamic radiation, anterior cingulum and sagittal stratum ~ ↑ depression severity.
		HC: 16.4 ± 1.4	HC (6M; 10F)		Clinical: KSADS, BDI, CDRS-R, MASC	
	[181]	MDD: 15.8 ± 1.4	MDD (8M; 11F)	Investigate sgACC FC in adolescent depression during negative emotional processing.	Imaging: fMRI	MDD: ↑ sgACC- amygdala Functional connectivity and ↓ sgACC-fusiform gyrus, sgACC-precuneus, sgACC-insula, and sgACC-middle frontal gyrus functional connectivity. ↓ sgACC-precuneus functional connectivity ~ ↑ depression severity.
		HC: 16.1 ± 1.2	HC (8M; 11F)		Clinical: BDI	
	[93]	HC: 16 ± 2.74	HC (6M; 7F)	Evaluate whether the observed WM disruptions are associated with increased vulnerability to psychopathology during prospective follow-up	Imaging: DTI	MDD at FUP ~ ↓ FA in the superior longitudinal fasciculi & the right cingulum-hippocampal Projection. SUD at FUP ~ ↓ FA in the right cingulum-hippocampal projection.
		MT: 15.89 ± 2.79	MT (5M; 14F)		Clinical: KSADS, FH-RDC, CDRS-R, HDRS, CGAS, BDI DUSI	
	[182]	OCD: 13.95 ± 2.52	OCD (9M; 16F)	Investigate the development of the ACC and its associations with psychopathology.	Imaging: fMRI	OCD: ↑ ACC activity during error responses in bilateral insular cortex during high conflict tasks
		HC: 13.71 ± 2.85	HC (9M; 16F)		Clinical: YBOCS, ADIS, CDI, STAI-C, CBCL	
	[81]	OCD: 13.78 ± 2.58	OCD (11M; 18F)	Identify differences in regional brain volume between medication-free pediatric OCD patients and controls and examine changes after cognitive behavioural therapy	Imaging: VBM	OCD: ↑ Orbitofrontal GMV after treatment ~ ↑ symptom improvement
		HC: 13.6 ± 2.73	HC (11M; 18F)		Clinical: YBOCS, ADIS, CDI, STAI-C, CBCL	
	[183]	BPD-I: 14.57 ± 1.98	BPD-I (11M; 7F)	Examine patterns of activity and connectivity in youth with BPD.	Imaging: fMRI	BP-I: ↑ activity in amygdala and VMPFC regulation regions to happy faces and reduced DLPFC activity to fearful faces compared to HC. BPD-NOS: ↓ PFC activity to neural faces compared to HC.
		BPD-NOS: 12.59 ± 2.27	BPD-NOS (11M; 7F)		Clinical: KSADS, MFQ, SCARED, CALS	
		HC: 13.67 ± 2.55	HC (7M; 11F)			
	[71]	HC: 23.9 ± 2.3	HC (12M; 21F)	Evaluate patterns of grey matter changes very early in the course of affective illness compared to those with discrete disorders and/or illness persistence	Imaging: sMRI	ST-2/3: ↓ GMV in frontal brain regions
		ST-1: 20.4 ± 5.2	ST-1 (8M; 15F)		Clinical: HDRS, SOFAS, BPRS	
		ST-2/3: 23.5 ± 3.5	ST-2/3 (14M; 10F)			
	[96]	HC: 23.82 ± 2.52	HC (15M; 24F)	Examine the association between microstructural WM changes and different stages of psychiatric illness.	Imaging: DTI	ST-2/3: ↓ FA within the left anterior corona radiata compared to HC.
		ST-1B: 21.36 ± 3.51	ST-1B (24M; 49F)		Clinical: HDRS, BPRS, SOFAS	
						ST-1B: pattern of ↓ FA within the left anterior corona radiate (less WM involvement than ST-2/3)
		ST-2/3: 22.45 ± 4.35	ST-2/3 (37M; 32)			
	[92]	BPD: 23.03 ± 5.04	BPD (23M; 35F)	Examine WM microstructural changes in BPD.	Imaging: DTI	BPD: ↓ FA in the genu, body and splenium of the corpus callosum as well as the superior and anterior corona radiata. ↑ radial diffusivity.
		HC: 24.05 ± 2.92	HC (12M; 28F)		Clinical: HDRS, YMRS, SOFAS, BPRS	
	[184]	OCD: 13.1 ± 2.7	OCD (7M; 5F)	Investigate possible regional brain dysfunction in premotor cortico-striatal activity, correlate brain activation with severity of obsessive-compulsive symptomatology; And, detect possible changes in brain activity after pharmacological treatment	Imaging: fMRI	OCD: ↑ activation bilaterally in the middle frontal gyrus. Clinical improvement following pharmacological treatment ~ ↓ activation in left insula and left putamen
		HC: 13.7 ± 2.8	HC (7M; 5F)		Clinical: ChIPS, Y-BOCS, CDI, STAI-C, LOI-CV	
	[98]	OCD: 12.5 ± 2.9	OCD (6M; 5F)	Measure neurometabolite concentrations in anterior cingulate-medial frontal cortex and right and left striatum of drug naïve children and adolescents with OCD	Imaging: MRS	OCD: ↓ total Cho in left striatum (this ↓ did not change over time and persisted at follow-up Assessment)
		HC: 14.5 ± 2.8	HC (5M; 7F)		Clinical: Y-BOCS, CDI, STAI-C, LOI-CV	
	[185]	BPD: 27 ± 10	BPD (26M; 32F)	Assess changes in GMV in BPD.	Imaging: sMRI	BPD: ↑ GMV in portions of the VLPFC and hippocamps complex. ↑ GMV in amygdala proper and caudate. ↑ number of depressive episodes ~ ↑ GMV in the right cingulate gyrus bilaterally and right thalamus and bilateral lenticulate nuclei, and left cerebellar vermis. ↑ illness duration ~ ↓ GMV in left cerebellar vermis.
		HC 27 ± 10	HC (21M; 27F)		Clinical: SCID, KSADS	
	[83]	OCD: 12.79 ± 2.64	OCD (10M; 21F)	Measure pituitary gland volume in OCD	Imaging: MRI	OCD: ↓ pituitary gland volume ~ ↑ compulsive symptom severity (more pronounced in males).
		HC: 12.89 ± 2.66	HC (10M; 21F)		Clinical: YBOCS, HAMA, HDRS	
	[74]	MDD: 8 – 17years	MDD (10M; 13F)	Examine temporal lobe anatomy in pediatric patients with MDD near the onset of illness before treatment	Imaging: MRI	MDD: ↑ left and right amygdala: hippocampus volume ratios ~ ↑ severity of anxiety (but not ↑ depression severity or duration of illness)
		HC: 8–17 years	HC (10M; 13F)		Clinical: CDRS-R, HAMA	
	[87]	ANX: 11.8 ± 1.8	ANX (6M; 6F)	Examine the relationships between pretreatment amygdala activity and treatment response in a sample of anxious children and adolescents	Imaging: fMRI	ANX: ↑ left amygdala activation pre-treatment ~ treatment response to CBT or medication. (no association between pre-treatment symptom severity and pre-treatment amygdala activity)
					Clinical: KSADS, CGI	
	[78]	SAD: 21.80 ± 3.68	SAD (14M; 6F)	Explore the GMD deficits in drug-naïve adult SAD patients	Imaging: VBM	SAD: ↓ GMD in bilateral thalami, right amygdala, and right precuneus. ↓ right amygdala GMD ~ ↑ disease duration and ↓ age of onset.
		HC: 21.58 ± 3.72	HC (13M; 6F)		Clinical: HAMA, HDRS, LSAS, SCID	
	[69]	DD: 15.4 ± 1.5	DD (3M; 23F)	Examine GMV in brain areas putatively involved in affective psychopathology.	Imaging: VBM	DD: ↓ bilateral dorsal ACC volume. No association with clinical severity of depression or anxiety.
		HC: 14.7 ± 1.5	HC (3M; 23F)		Clinical: ADIS, CDI, RCADS, YSR, CBCL	
	[100]	BPD: 15.5 ± 1.5,	BPD (5M; 23F)	Compare in vivo neurometabolite concentrations in bipolar adolescents with a depressed episode	Imaging: MRS	BPD: ↑ NAA in the ACC and VLPFC. ↑ Cho and Cr in the VLPFC.
		HC: 14.6 ± 1.8	HC (4M; 6F)		Clinical: KSADS, CDRS-R	
	[186]	BPD: 14.3 ± 1.1	BPD (6M; 11F)	Investigate the effects of pharmacotherapy on brain function underlying affect dysregulation and cognitive function in pediatric bipolar disorder.	Imaging: fMRI	BPD: YMRS improvement ~ ↓ VMPFC activity. Normalization of activity in the inferior frontal gyrus following pharmacological treatment.
		HC: 14.1 ± 2.4	HC (7M; 7F)		Clinical: YMRS, KSADS, CDI, CDRS-R	
	[187]	BPD:	BPD (16M; 8F)	Determine the relative effects of risperidone and divalproex on brain function in pediatric mania	Imaging: fMRI	BPD: Divalproex treatment ~ ↑ activity in left MPFC relative and modulation of positive emotions to risperidone. ↑ pre-treatment right amygdala activity with negative and positive condition in the risperidone group, and left amygdala with positive condition in divalproex group predicted poor response on YMRS.
		HC: 13.9 ± 3.4	HC (7M; 7F)		Clinical: KSADS, CDRS, YMRS	
	[86]	gSP: 25.91 ± 5.50	gSP (8M; 13F)	Examine the change in amygdala-insula-medial frontal function during perception of social threat cues before and after SSRI treatment	Imaging: fMRI	gSP: SSRI treatment ~ ↓ amygdala reactivity to fearful faces (which was ↑ pre-treatment) and ↑ ventral MPF activity to angry faces (which was ↓ Pre-treatment treatment). No correlations with symptom improvement.
		HC: 26.95 ± 8.11	HC (10M; 9F)		Clinical: SCID, LSAS, HDRS, BDI, STAI	
	[188]	OCD: 28.8 ± 8.2	OCD (4M; 5F)	Identify neuroimaging predictors of medication response in contamination-related obsessive compulsive disorder OCD	Imaging: PET	OCD: ↓ rCBF in OFC and ↑ rCBF values in PCC predicted better fluvoxamine treatment response.
					Clinical: AAS, OCDAS	
	[70]	HC: 17.19 ± 1.87	HC (7M; 9F)	Investigate the role of dysregulation of frontal-limbic circuits in the symptomology of this disorder	Imaging: sMRI	MDD: ↑ right and left rostral MFG, and left caudal anterior cingulate cortex thickness. ↑ age ~ ↓ left MFG thickness.
		MDD: 16.89 ± 2.01	MDD (9M; 21F)		Clinical: CDRS, KSADS, BDI	
	[84]	OCD: 12.70 ± 3.11	OCD (13M; 8F)	Investigate the regional morphology of the CC in OCD.	Imaging: sMRI	OCD: ↑ corpus callosum (except the isthmus). ↑ CC area, genu, anterior body, posterior body, isthmus and anterior splenium ~ ↑ compulsive symptom severity
		HC: 12.74 ± 3.12	HC (13M; 8F)		Clinical: YBOCS, HAMA, HDRS, KSADS	
	[79]	OCD: 12.89 ± 3.23	OCD (5M; 6F)	Evaluate neuroanatomic changes in the thalamus of OCD patients near illness onset before and after cognitive behavioral therapy	Imaging: sMRI	OCD: No significant change in thalamic volume after CBT
					Clinical: YBOCS, HDRS, HAMA, KSADS	
	[73]	MDD: 15.35 ± .34,	MDD (3M; 17F)	Examine amygdala and hippocampus volumes in pediatric MDD.	Imaging: sMRI	MDD: ↓ left and right amygdala volumes. No correlations with symptom severity, age of onset or illness duration.
		HC: 14.08 ± .31	HC (8M; 16F)		Clinical: HDRS, FH-RDC, KSADS	
	[75]	GAD: 22.9 ± 4.1,	GAD (16F)	Investigate the neural substrates associated with excessive and persistent worrying in GAD	Imaging: sMRI	GAD: ↑ amygdala and DMPFC volumes. ↑ symptom severity ~ ↑ DMPFC and ACC volumes
		HC: 23.7 ± 3.7	HC (15F)		Clinical: SCID, BDI, MCQ	
	[189]	BPD: 14.6 ± 2.2	BPD (11M; 12F)	Examine the neurofunctional effects of ziprasidone in manic adolescents	Imaging: fMRI	BPD: Ziprasidone treatment ~ ↑ in right BA 11 and 47 activation. No association with symptom improvement. ↓ BL right BA 47 activation ~ ↑ improvement of YMRS score.
		HC: 15.0 ± 1.8	HC (6M; 4F)		Clinical: YMRS, CGI, KSADS	
	[101]	BPD-R: 15.4 ± 1	BPD-R (4M; 3F)	Evaluate the in vivo effects of extended-release divalproex sodium on the glutamatergic system in adolescents with BPD and neurochemical predictors of clinical remission.	Imaging: MRS	BPD-r: ↓ BL Glx in LVLPFC. Change in LVLPFC Glu ~ change in YMRS score
		BPD-NR: 14.1 ± 2.2	BPD-NR (6M; 1F)		Clinical: KSADS, CDRS, CGI, YMRS	
		HC: 14.4 ± 1.6	HC (6M; 9F)			
	[88]	BPD-RE: 13.5 ± 2.4	BPD-RE (13M; 9F)	Determine functional connectivity among patients with pediatric BPD who are responders to pharmacotherapy and those who are nonresponders,	Imaging: fMRI	BPD-RE: ↑ connectivity of the amygdala before and after treatment compared to BPD-NRE. ↑ right amygdala functional connectivity after treatment ~ ↑ improvement in mania symptoms
		BPD-NRE: 13.3 ± 2.0	BPD-NRE (6M; 6F)		Clinical: KSADS, YMRS, CDRS-R	
		HC: 14.2 ± 3.1	HC (7M; 7F)			
	[72]	MDD: 28.8 ± 10.7	MDD (35M; 30F)	Evaluate the early effects of antidepressant therapy, as well as of key clinical variables, on ACC volume	Imaging: sMRI	MDD: >3 untreated depressive episodes ~ ↓ subcallosal gyrus volumes compared to HC.
		HC: 28.4 ± 10.7	HC (37M; 56F)		Clinical: YMRS, GAF, HDRS, SCID	
	[82]	OCD: 16.6 ± 1.5	OCD (14M; 12F)	Identify structural GM and WM microstructure changes in pediatric OCD	Imaging: sMRI, DTI	OCD: ↑ symptom severity ~ ↑ GM volume in right insula, posterior orbitofrontal cortex, brainstem and cerebellum,
		HC: 16.5 ± 1.4	HC (14M; 12F)		Clinical: YBOCS	
	[80]	OCD: 22.0 ± 5.2	OCD (3M; 5F)	Evaluated resting brain metabolism and treatment response in OCD patients.	Imaging: PET, MRI	OCD: ↑ clinical improvement ~ ↑ changes in bilateral dosal ACC and in the right middle occipital gyrus
		HC: 21.5 ± 5.9	HC (8F)		Clinical: YBOCS, HDRS	

Note. *Sample*: *ADHD* attention deficit hyperactivity disorder, *ANX* anxiety disorder, *AUD* alcohol use disorder, *BPD* bipolar disorder, *BPD-I* bipolar disorder I, *BPD-O* bipolar disorder with obesity, *BPD-L* bipolar disorder with low alcohol use, *BPD-H* bipolar disorder with high alcohol use, *BPD-N* bipolar disorder without obesity, *BPD-NOS* bipolar disorder not otherwise specified, *BPD-R* bipolar disorder remitters, *BPD-NR* bipolar disorder non remitters, *BPD-RE* bipolar disorder responders to pharmacotherapy, *BPD-NRE* bipolar disorder non responders to pharmacotherapy, *DD* depressive disorder, *CU* cannabis user, *GAD* generalised anxiety disorder, *gSP* generalised social phobia, *HC* healthy controls, *HC-O* healthy controls with obesity, *HC-N* healthy controls without obesity, *MDD* major depression disorder, *MDDx* major depression disorder with borderline personality disorder, *MHP* mental health patients (mixed diagnosis sample), *MT* childhood maltreatment, *OCD* obsessive compulsive disorder, *PC* psychiatric control (i.e. psychiatric diagnosis but no suicide attempt), *PSD* psychotic spectrum disorder, *SA* suicide attempters, *ST* stage of illness; 1B, 2, & 3, *SAD* social anxiety disorder

*Measures*: *AAS* anxiety analogue scale, *ACQ* alcohol consumption questionnaire, *ADIS* anxiety disorders interview schedule, *ASI* addictions severity index, *AUDIT* alcohol use disorder identification test, *BDI* beck depression inventory, *BMI* body mass index, *PRS* brief psychiatric rating scale, *CALS* child affect liability scale, *CBCL* child behaviour checklist, *CDI* children’s depression inventory, *CDRS* children’s depression rating scale; *R* revised, *CGAS* children’s global assessment scale, *CGI* clinical global impression scale, *ChIPS* children’s interview for psychiatric syndromes, *CDDR* customary drinking and drug use record, *CGAS* child global assessment scale, *C-CASA* Columbia Classification Algorithm of Suicide Assessment, *CSHF* Colombia Suicide History Form, *DTI* diffuse tensor imaging, *DUSI* drug use screening inventory, *FH-RDC* family history-research diagnostic criteria, *fMRI* functional magnetic resonance imaging, *GAF* global assessment of functioning, *HAMA* Hamilton anxiety rating scale, *HDRS* Hamilton depression rating scale, *K-SADS* kiddie schedule for affective disorders and schizophrenia, *LHAUD* lifetime history of alcohol use disorder, *LIFE* modified longitudinal interval follow-up examination, *LOI-CV* Leyton Obsessive Inventory-Child Version, *LSAS* Liebowitz social anxiety scale, *MASC* multidimensional anxiety scale for children, *MCQ* meta cognition questionnaire, *MFQ* Mood frequencies questionnaire, *MRS* magnetic resonance spectroscopy, *PET* positron emission tomography, *PRS* Pfeffer rating scale, *OCDAS* obsessive compulsive disorder analogue scale, *RCADS* the revised child anxiety and depression scale, *SAC* substance abuse course-modified life II, *SCARED* screen for child anxiety related disorders, *SCID* structured clinical interview for DSM, *SIQ* suicide ideation questionnaire, *SIS* suicide intent scale, *SOFAS* social and occupational functioning assessment scale, *sMRI* structural magnetic resonance imaging, *STAI-C* state- trait anxiety inventory – child version, *TLFB* time-line followback, *VBM* voxel-based morphometry, *Y-BOCS* Yale–Brown obsessive-compulsive scale, *YMRS* young mania rating scale, *YSRS* the youth self-report scale

*Findings*: ↑ = Increased, Improved or Higher, ↓ = Decreased, Reduced or Lower, ~ = ‘is associated with’, *ACC* anterior cingulate cortex, *AD* Axial diffusivity, *ACG* Anterior Cingulate Gyrus, *BA* Broadman Area -24, *BL* baseline, *CV* cerebellar vermis, *DmPFC* dorsomedial prefrontal cortex, *DWMH* deep white matter hyperintensities, *FA* fractional anisotropy, *GABA* gamma-aminobutyric acid, *GM* grey matter, *GMV* grey matter volumes, *GSH* glutathione, *iFC* intrinsic functional connectivity, *LFG* left fusiform gyrus, *MD* mean diffusivity, *MFG* middle frontal gyus, *MPFC* medial prefrontal cortex, *OFC* orbitofrontal cortex, *RD* radial diffusivity, *PVH* periventricular hyperintensities, *PCG* precentral Gyrus, *PFC* prefrontal cortex, *TBV* total brain volumes, *TLV* temporal lobe volume, *VMPFC* ventromedial prefrontal cortex, *WMH* white matter hyperintensities, *WMV* White Matter Volumes

*indicates that the study features more than once in the data synthesis

**Table 5:** Sleep-wake and circadian biology studies evaluating the five functional domains in young people (12-30 yrs) with a mood and/or anxiety disorder

Outcome measure	Study	Age (mean ± SD)	Sample (N)	Aims	Key measures	Key findings
*Social and economic participation*	[103]	PD: 30.6 ± 6.1	PD (8M; 12F)	Determine whether HPA activity can predict FUP functional status.	SWC: 24-hour cortisol samples, ACTH profiles, CRH stimulation test	PD: ↑ cortisol secretion pre-treatment ~ ↓ social and economic participation (better than pre-treatment clinical severity)
					Functional: SDS	
	[102]*	MDD (M): 12.8 ± 2.6, MDD (F): 13.6 ± 1.9	MDD (22M; 33F)	Investigate whether diurnal changes in cortisol and DHEA levels are associated with the occurrence of undesirable life events.	SWC: Cortisol/DHEA ratio,	MDD: ↑ cortisol/DHEA ratios at BL ~ ↓ social and economic participation at FUP.
					Functional: Semi-structured interview	
	[104]	MHP: 12.1 (7 – 17.9 years)	MHP (62M; 40F)	Investigate whether cortisol reactivity is associated with internalizing problem behaviour	SWC: Cortisol level	MHP: ↑ cortisol secretion during the social interaction task ~ ↓ social and economic participation
					Functional: CBCL, SASC, CDI	
*Suicide and self-harm *	[105]*	MDD: 25.19 ± 2.42	MDD (33M; 23F)	Examine baseline neuroendocrine predictors of follow up clinical features	SWC: Sleep EEG, GH secretion, blood cortisol	MDD: ↑ BL GH secretion during first 4 hours of sleep ~ a suicide attempt during FUP
		HC: 25.92 ± 2.16	HC (10M; 11F)			
					Functional: Clinical interview	
	[106]	MDD: 25.19 ± 2.42	MDD (33M; 23F)	Assess whether any premorbid cortisol abnormalities were associated with depressive course of illness	SWC: Sleep EEG, GH secretion, blood cortisol	MDD: ↑ BL cortisol secretion in the late evening hours ~ suicide attempts during FUP
		HC: 25.92 ± 2.16	HC (10M; 11F)			
					Functional: Clinical interview	
	[107]*	MDD: 16 ± 0.3	MDD (6M; 14F)	Compare sleep EEG profiles of a sample of outpatient adolescents	SWC: Sleep EEG, blood samples	MDD: ↓ Delta sleep variable ~ ↑ suicidality (and depression severity).
		HC: 15.6 ± 0.6	HC (7M; 6F)			
					Functional: HDRS	
*Clinical syndrome*	[108]	CS: 17.04 ± 0.36	CS (57M; 173F)	Examine whether individual differences in the CAR serve as a premorbid risk factor for MDD	SWC: Salivary cortisol	CS: ↑ cortisol after waking at BL ~ ↑ risk of developing MDD at FUP
					Clinical: SCID, LSI	
	[118]	HYP: 20.91 ± 3.72	HYP (8M; 23F)	Assess circadian activity and sleep in individuals at behavioral high-risk of hypomania/bipolar disorders	SWC: Actigraphy	HYP: ↑ variability in duration, fragmentation and efficiency of sleep, ↓ sleep duration and later more variable be times.
		HC: 22.12 ± 2.83	HC (8M; 16F)		Clinical: SCID, HPS, HIQ, ISS	
	[120]	MDD: 12 ± 1.9	MDD (2M; 4F)	Explore the effects of fluoxetine on sleep EEG	SWC: Sleep EEG	MDD: ↑ stage 1 sleep, arousals and REM density ~ fluoxetine treatment
					Clinical: K-SADS, CDRS, BDI, WSAS	
	[105]*	MDD: 25.19 ± 2.42	MDD (33M; 23F)	Examine baseline neuroendocrine predictors of follow up clinical features	SWC: Sleep EEG, GH secretion, blood cortisol	MDD: Premorbidly, earlier and more steep GH secretion at sleep onset
		HC: 25.92 ± 2.16	HC (10M; 11F)			
					Clinical: Clinical interview	
	[114]	MDD: 17.04 ± 0.35	MDD (4M; 7F)	Examine the associations between MDD and anxiety disorders, and HPA- axis functioning	SWC: Salivary cortisol	P-MDD & MDD/ANX: flatter diurnal cortisol slopes
		ANX: 17.04 ± 0.37	ANX (8M; 21F)		Clinical: MASQ, LSI	
		MDD/ANX: 16.85 ± 0.21	MDD-ANX (4M; 8F)			
		P-MDD: 17.13 ± 0.37	P-MDD (11M; 45F)			
		P-ANX: 17.02 ± 0.38	P-ANX (6M; 2F)			
	[109]	HR: 16.8 ± 1.7	HR (14M; 15F)	Examine the cortisol increase after awakening and basal cortisol levels hypothesis that high-risk offspring are more reactive to psychosocial stress than low-risk offspring	SWC: Salivary cortisol	HR: ↑ daytime cortisol in their natural environment.
		LR: 16.6 ± 2.1	LR (14M; 15F)		Clinical: CDI, CBCL, PANAS	
	[110]	HR: 18.3 ± 2.6	HR (12M; 12F)	Determine whether HR individuals exhibit elevated cortisol levels relative to LR individuals during two weeks of daily sampling	SWC: Salivary cortisol	HR: ↑ afternoon cortisol levels in their natural environment
		LR: 18.0 ± 2.3	LR (11M; 11F)		Clinical: BDI, CDI, PSWQ, CBCL, RLEQ	
	[102]*	MDD (M): 12.8 ± 2.6	MDD (22M; 33F)	Investigate whether diurnal changes in cortisol and DHEA levels are associated with the occurrence of undesirable life events.	SWC: Cortisol/DHEA ratio,	MDD: ↑ cortisol/DHEA ratios at BL ~ persistent major depression at FUP
		MDD (F): 13.6 ± 1.9			Clinical: Semi-structured interview	
	[113]	Mild: 14.73 ± 2.30 Moderate: 15.69 ± 1.58	Mild (10M; 20F) Moderate (7M; 9F)	Examine cortisol reactivity to a psychological stress challenge in depressed adolescents.	SWC: Salivary cortisol	Moderate/severe depression: ↓ cortisol response regardless of child maltreatment history
					Clinical: CECA, BDI-II, K-SADS	
		Severe: 16.00 ± 2.00	Severe (6M; 19F)			
	[119]	MDD: 23.94 ± 2.31	MDD (8M; 9F)	Investigate the effect of reducing slow waves during sleep on depression symptomology	SWC: Sleep EEG	MDD: ↑ overnight dissipation of SWA predicted ↓ in depressive symptoms.
					Clinical: QIDS, HDRS	
	[107]*	MDD: 16 ± 0.3	MDD (6M; 14F)	Compare sleep EEG profiles of a sample of outpatient adolescents	SWC: Sleep EEG, blood samples	MDD: ↓ Delta sleep variable ~ ↑ depression severity.
		HC: 15.6 ± 0.6	HC (7M; 6F)			
					Clinical: HDRS	
	[117]	DD: 15.35 ± 1.85	DD (18M; 28F)	Assess sleep disturbances pain and pubertal development in adolescent depressive disorders	SWC: Actigraphy	DD: ↓ sleep efficiency and total time asleep, ↑ time awake after sleep onset. ↑ pain intensity and depressive symptoms predicted worse sleep quality
		HC: 14.83 ± 1.76	HC (17M; 43F)		Clinical: K-SADS, PDS, CES-D, BPD	
	[111]	MDD: 22.4 ± 1.5	MDD (9M; 17F)	Examine the relationship between longitudinal clinical course, sleep and cortisol in adolescent depression	SWC: Sleep EEG	MDD: recurrent illness ~ ↑ plasma cortisol near sleep onset at BL.
		HC: 21.9 ± 1.7	HC (13M; 20F)		Clinical: K-SADS	
						HC: high density REM and ↓ REM latency at BL ~ the development of depression a FUP
	[112]	MDD: 15.6 ± 1.4	MDD (6M; 10F)	Examine EEG sleep and HPA changes during MDD episodes and recovery	SWC: NUFC, sleep EEG	MDD: ↓ NUFC excretion during remission
		HC: 15.8 ± 1.9	HC (7M; 9F)		Clinical: PRS, HDRS, K-SADS	
	[115]	UPD: 21.8 ± 4.3	UPD (5M; 13F)	Evaluate the potential of circadian measures as early markers of mood disorders subtypes	SWC: Actigraphy, DLMO	BPD:↓ and later onset of melatonin secretion
		BPD: 22.8 ± 4.8	BPD (3M; 11F)		Clinical: Psychiatric interview (DSM-IV criteria), BDI	
	[116]	HC: 24.8 ± 2.5	HC (8M; 12F)	Investigate objectively the 24-h sleep–wake cycle in adolescents and young adults with mood disorders	SWC: Actigraphy	BPD: 62 % had delayed sleep (during a depressive phase), and later sleep offset compared to UPD and HC
		UPD: 20.1 ± 4.7	UPD and BPD (28M; 47F)		Clinical: Psychiatric interview (DSM-IV criteria)	
		BPD: 23.2 ± 4.3				
						UPD: 30 % had delayed sleep
						HC: 10 % had delayed sleep
	[121]	Stage 1a: 17.6 ± 4.0	Stage 1a (7M; 11F)	Determine if disturbed sleep–wake cycle patterns in young people with emerging mental disorder are associated with stages of illness	SWC: Actigraphy	Stage 1b & 2: ↑ delayed sleep schedule, especially on weeknights
		Stage 1b: 19.1 ± 4.1	Stage 1b (44M; 38F)		Clinical: Psychiatric interview (DSM-IV criteria)	
		Stage 2+: 22.4 ± 4.3	Stage 2+ (27M; 27F)			Stage 1a & 2+: ↓ sleep efficiency
		HC: 24.4 ± 3.1	HC (11M; 12F)			

Note. Sample: ANX anxiety disorder, BPD bipolar disorder, CS community sample, DD depressive disorder, HC healthy controls, HR high risk participants (offspring of parents with bipolar disorder), HYP hypomanic participants, LR low risk participants (offspring of parents without a mental disorder), MDD-ANX comorbid Major depressive disorder and anxiety disorder, MDD major depression disorder, MHP mental health patients (mixed diagnosis sample), P-MDD past major depressive disorder, P-ANX past anxiety disorder, PD panic disorder

Measures: ACTH adrenocorticotropic hormone, BDI beck depression inventory, BPD body pain diagram, CBCL child behaviour checklist, CDI children’s depression inventory, CDRS children’s depression rating scale, CECA childhood experience of case and abuse contextual semi-structured interview and rating system, CES-D Center for Epidemiologic Studies Depression, CRH corticotropin-releasing hormone, DHEA dehydroepiandrosterone, DLMO dim light melatonin onset, DSM-IV diagnostic and statistical manual of mental disorders IV, EEG electroencephalography, GH growth hormone, HDRS Hamilton depression rating scale, HPS hypomanic personality scale, HIQ hypomanic interpretations questionnaire, ISS internal state scale, K-SADS schedule for affective disorders and schizophrenia for school age children, LSI life stress interview, MASQ mood and anxiety symptom questionnaire, NUFC nocturnal urinary free cortisol, PANAS positive and negative affect scale, PDS pubertal developmental scale, PRS Pfeffer rating scale, PSWQ Penn state worry questionnaire, QIDS quick inventory of depressive symptomatology, RLEQ recent life events questionnaire, SASC social anxiety scale for children, SCID structured clinical interview for DSM, SDS Sheehan disability scale, SWC sleep-wake and circadian biology, WSAS work and social adjustment scale

Findings: ↑ = Increased, Improved or Higher, ↓ = Decreased, Reduced or Lower, ~ = ‘is associated with’, BL baseline, FUP follow-up, NUFC nocturnal urinary free cortisol, REM rapid eye movement, SWA slow wave activity

*indicates that the study features more than once in the data synthesis

**Table 6:** Neurophysiological studies evaluating the five functional domains in young people (12-30 yrs) with a mood and/or anxiety disorder

Outcome measure	Study	Age (mean ± SD)	Sample (N)	Aims	Key measures	Key findings
*Social and economic participation*	[190]	MHP: 22.1 ± 4.0	BPD (18)	Determine the longitudinal relationship between MMN/P3a and functional outcomes in patients.	Nα: MMN	BPD & PSD: ↑ BL MMN ~ ↑ social and economic participation at FUP
			PSD (13)		Functional: SOFAS, WHO-DAS-II	
*Physical health*	[191]*	MDD: 17.1 ± 0.6	MDD (8F)	Investigate the effect of nicotine on resting EEG activity and affect.	Nα: EEG	MDD: ↓ rPR theta & ↓ smoking withdrawal, craving and physical symptoms ~ acute nicotine administration.
					Functional: HONC	
*Suicide and self-harm*	[122]	SA: 29.5 ± 13.3, HC: 34 ± 13.3	SA (24M; 16F)	Investigate the trait predisposing to DSH by examining EEG and peripheral monoamine activity.	Nα: EEG and blood samples	SA: ↓ CNV and whole blood 5-HT ~ multiple episodes of self-harm.
			HC (13M; 14F)		Functional: HLS, MADRS, SIS	
	[123]	SA: 14 (12 – 17yrs)	SA (16F)	Examine EEG alpha asymmetry among high-risk adolescents	Nα: EEG alpha asymmetry	SA: ↑ posterior alpha asymmetry ~ suicidal intent (not depression severity)
		HC: 14 (12 – 17yrs)	HC (22F)		Functional: HASS, SIS	
	[124]	rMDD + CSA: 31.60 ± 10.98	rMDD + CSA (15F)	Examine the association between CSA, MDD and maladaptive behaviour.	Nα: EEG	rMDD + CSA: ↑ subgenual ACC activation during reward based decision making, ↓ reaction time during incentive-based trials ~ ↑ frequency of self harm/suicidal behaviours.
		rMDD: 24.81 ± 3.94	rMDD: (16F)		Functional: YRBS (adult version)	
		HC: 30.44 ± 10.78	HC (18F)			
*Alcohol and substance use*	[192]	BPD-L: 21.8 ± 3.9	BPD-L (5M; 11F)	Investigate the effects of alcohol use on MMN in BP.	Nα: MMN	BPD-H: ↓ temporal MMN
		BPD-H: 22.6 ± 3.4	BPD-H (9M; 17F)		Functional: AUDIT	
		HC-L: 22.4 ± 2.6	HC-L (6M; 14F)			
		HC-H: 23.4 ± 3.2	HC-H (6M; 8F)			
	[193]	AD: 24 ± 3.77	AD (44M; 47F)	Explore the use of a startle paradigm and its association with alcohol use.	Nα: Startle, ERP	AD: ↑ facilitation, ↓ inhibition of the N4S component by pre pulse stimuli.
		BD: 24.6 ± 5.76	BD (23M; 18F)		Functional: SSAGA, FHAM	
		AFF: 22.9 ± 3.94	AFF (32M; 65F)			
		DD: 23.5 ± 3.17	DD (51M; 61F)			
*Clinical syndrome*	[128]	ANX : 12.9 ± 2.6	ANX (7M; 13F)	Examine the relationship between ASR, symptom reduction and treatment success.	Nα: Multiple muscle ASR	ANX: ↓ in multiple muscle ASR ~ ↓ in anxiety symptoms.
		HC: 12.0 ± 2.5	HC (10M; 15F)		Clinical: ADIS-C/P, SCAS	
						ANX: ↑ multiple muscle ASR predicted CBT treatment response
	[133]	OCD: 13.9 ± 2.4	OCD (18M; 22F)	Assess ERN as a biomarker for OCD	Nα: ERN	OCD & SIB: ↑ ERN at Cz (independent of symptom severity, current diagnostic status and treatment effects).
		SIB: 13.9 ± 2.4	SIB (13M; 6F)		Clinical: Y-BOCS, CBCL, MASC, CDI	
		HC: 13.8 ± 2.3	HC (20M; 20F)			
	[134]	ANX: 11.8 ± 2.3	ANX (3M; 10F)	Demonstrate ERN amplitude is increased in young anxiety patients.	Nα: ERN	ANX & OCD: ↑ ERN at Cz (independent of symptom severity, current diagnostic status and treatment effects).
		OCD: 12.7 ± 2.2	OCD (8M; 18F)		Clinical: Y-BOCS, CBCL, MASC, CDI	
		HC: 12.4 ± 2.2	HC (14M; 13F)			
	[194]	RES: 14.1 ± 2.8	RES (2M; 6F)	Examine the relationship between TMS with subsequent treatment response	Nα: TMS	NoRES: ↑ deficits in pre-treatment LICI
		NoRES: 13.1 ± 1.6	NoRES (5M; 3F)		Clinical: CDRS-R, QIDS, CGI-severity scale	
	[195]	HC: 25.54 ± 3.41	HC (28M; 16F)	Investigate the intensity evaluation of social stimuli in depression	Nα: ERP (N170, P1, P2)	MDD: ↑ intensity scores for sad faces compared with HC, ↑ reaction times for all faces and ↑ P1 & P2 amplitude for sad faces
		DEP: 25.96 ± 4.58	DEP (9M; 15F)		Clinical: SCID, BDI, HDRS, BAI	
		MDD: 26.58 ± 4.16	MDD (10M; 14F)			
						DEP: ↓ scores for happy and neutral faces, ↑ reactions times and ↑ P1 & P2 amplitude for happy faces compared to sad faces.
	[196]	HC: 27.7 ± 7.0	HC (14M; 12F)	Assess brain function impairments in bipolar patients.	Nα: Resting EEG	BPD: ↑ power in all wave bands. Marked increases in right temporal theta and left occipital beta.
		BPD: 30.7 ± 6.1	BPD (10M; 19F)		Clinical: BDI	
	[135]	OCD: 13.3 ± 2.8,	OCD (13M; 5F)	Examine ERN in paediatric patients with OCD	Nα: ERN	OCD: ↑ ERN pre-treatment and after treatment. No relationship with symptom severity or changes in symptom severity
		HC: 11.9 ± 2.6	HC (8M; 10F)		Clinical : Y-BOCS	
	[197]	HC: 17 ± 1.6	HC (43F)	Evaluate the effects of depression and a family history of alcohol or substance dependence on P300.	Nα: ERP (P300)	DD: ↓ P300 amplitude. No effect of family history of alcohol or drug dependence.
		HC-FHA: 16.5 ± 1.3	HC-FHA (31F)		Clinical: SSAGA, MAST, PANAS	
		HC-FHD: 16.1 ± 1.5	HC-FHD (27F)			
		DEP: 17.2 ± 1.4	DD (12F)			
		DEP-FHA: 17.3 ± 1.5	DD-FHA (9F)			
		DEP-FHD: 16.3 ± 1.3	DD-FHD (8F)			
	[191]*	MDD: 17.1 ± 0.6	MDD (8F)	Investigate the effect of acute nicotine administration on resting EEG activity and affect	Nα: EEG	MDD: Nicotine ↓ theta amplitude in right parietal region. No associations with mood.
					Clinical: BDI, HONC, PANAS	
	[129]	MDD: 30.4 ± 11.8	MDD (28M; 23F)	Assess the utility of baseline LDAEP predicting response to antidepressants.	Nα: LDAEP	MDD: steep N1 sLORETA-LDAEP at BL ~ treatment response. ↑ P2 sLORETA-LDAEP slope at week 1 ~ treatment response.
					Clinical: HDRS, MADRS	
	[167]*	OCD: 27 ± 9.8	OCD (15M; 16F)	Characterize the cognitive functions of the patients with OCD by utilizing ERPs and neuropsychological tests	Nα: ERP (P300)	OCD: ↓ P300 duration. ↓ stroop duration ~ ↑ P300 amplitude in occipital, parietal and temporal anterior regions.
		HC: 27.4 ± 9.1	HC (14M; 16F)		Clinical: HDRS	
	[52]*	OCD: 24.06 ± 5	OCD (21M; 9F)	Assess the relationship between cognitive dysfunction, clinical status and severity in OCD.	Nα: ERP (N100, P200, N200, P300)	OCD: ↑ P200 amplitude, unrelated to neither severity nor chronicity of illness. ↓ N200 amplitude (worsens with ↑ severity). ↓ N100 and P200 ~ ↑ chronicity
		HC: Matched	HC (21M; 9F)			
					Clinical: YBOCS	
	[136]	OCD-U: 25 ± 8.0	OCD-U (9M; 10F)	Examine the effects of chronic medication on error responses in OCD.	Nα: ERN	OCD: ↑ ERN, irrespective of medication use.
		OCD-M: 30.8 ± 9.5	OCD-M (9M; 10F)		Clinical: HDRS, HAMA, YBOCS	
		PC-M: 31.7 ± 10.6	PC-M (8M; 11F)			HC & PC: ↑ anxiety and depression ~ ↑ ERN amplitude
		HC: 25.3 ± 7.5	HC (11M; 10F)			
	[127]	DEP: 20.9 ± 0.55	DEP (515)	Examine whether recurrent major depression is associated with abnormal startle	Nα: ASR	DEP: ↑ ASR was associated with multiple (more than 1) depressive episode.
					Clinical: SCID	

Note. Sample : AFF affective disorder (not specified), AD alcohol dependence, ANX anxiety disorder, BD behavioural disorder, BPD bipolar disorder, BPD-L bipolar disorder with low alcohol use, BPD-H bipolar disorder with high alcohol use, DD depressive disorder, DD-FHA depressive disorder with family history of alcohol dependence, DD-FHD depressive disorder with family history of drug dependence, DrDep drug dependence, HC healthy controls, HC-FHA healthy control with family history of alcohol dependence, HC-FHD healthy control with family history of drug dependence, HC-L health control with low alcohol use, HC-H healthy control with high alcohol use, MDD major depression disorder, MHP mental health patients (mixed diagnosis sample), NoRES treatment non responders, OCD obsessive compulsive disorder, OCD-M obsessive compulsive disorder patient medicated, OCD-U obsessive compulsive disorder patients unmedicated, PC-M psychiatric control patient medicated, PSD psychotic spectrum disorder, RES treatment responders, rMDD remitted major depression disorder, rMDD+CSA remitted major depression disorder with childhood sexual abuse history, SA suicide attempters, SIB suicide ideation behaviour

Measures : ADIS-C/P anxiety disorders interview schedule for children, ASR auditory startle reflex, AUDIT Alcohol Use Disorder Identification Test, BAI beck anxiety inventory, BDI beck depression inventory, CBCL child behaviour checklist, CDI children’s depression inventory, CDRS children’s depression rating scale, CGI clinical global impression scale, EEG electroencephalography, ERP event related potential, ERN event related negativity, FHAM family history assessment module, HAMA Hamilton anxiety rating scale, HASS Harkavy Asnis suicide scale, HDRS Hamilton depression rating scale, HLS beck hopelessness scale, HONC hooked on nicotine checklist, LDAEP loudness dependant auditory evoked potential, MASC multidimensional anxiety scale for children, MADRS Montgomery-Asberg depression rating scale, MMN mismatch negativity, MAST Michigan Alco- holism Screening Test, PANAS positive and negative affect scale, QIDS quick inventory of depressive symptomatology, SCID structured clinical interview for DSM, SCAS Spence children’s anxiety scale, SIS suicide intent scale, SOFAS social and occupational functioning assessment scale, SSAGA semi-structured assessment for the genetics of alcoholism, TMS transcranial magnetic stimulation, WHO-DAS-II World Health Organisation Disability Assessment Scale II, Y-BOCS, Yale–Brown obsessive-compulsive scale, YRBS youth risk behaviour survey

Findings : ↑ = Increased, Improved or Higher, ↓ = Decreased, Reduced or Lower, ~ = ‘is associated with’, 5-HT serotonin, BL baseline, CBT cognitive behaviour therapy, CNV contingent negative variation, FUP follow-up, N4S late wave frontal ERP component responses, rPR right Parietal Region, RT reaction time

* indicates that the study features more than once in the data synthesis

**Table 7:** Metabolic studies evaluating the five functional domains in young people (12-30 yrs) with a mood and/or anxiety disorder

Outcome measure	Study	Age (mean ± SD)	Sample (N)	Aims	Key measures	Key findings
*Social and participation*	[140]*	MHP: 28.74 ± 10.38	MHP (38M; 28F)	Identify changes in the rates of obesity in never-treated patients with mood disorder over 4 years of follow-up.	Metabolic: BMI	MHP: ↑ BMI ~ ↑ social and economic participation
			(40 MDD, 26 BPD)		Functional: GAF	
*Physical health*	[198]	FH+: 18.9 ± 1.0	FH+ (32M, 53F)	Determine whether young people with a family history of depression have altered metabolic markers.	Metabolic/Functional: glucose, lipids and high-sensitivity CRP. BP, arterial stiffness and waking cortisol concentration.	FH+: ↑ peripheral and central BP, arterial stiffness and ↓ insulin sensitivity
		HC: 19.1 ± 0.1	HC (27M; 42F)			
*Suicide and self-harm*	[138]	SUC: 15.93 ± 1.48	SUC (15M; 32F)	Examine the relationship between serum cholesterol levels and suicidal behaviours	Metabolic: blood serum samples	SUC: ↑ cholesterol ~ current suicide behaviour (within the SUC group, ↑ serum cholesterol ~ ↓ severity of SUC, but not ~ symptom severity)
		PC: 16.22 ± 1.95	PC (58M; 47F)		Functional: SPI	
		PD: 25.3 ± 3.3	PD (37M; 35F)	Elucidate the relationships between alexithymia, suicide ideation and serum lipid levels.	Metabolic: BMI, blood serum samples	PD: ↓ HDL-C and ↑ VLDL-C ~ higher suicide ideation
					Functional: SSI	
	[137]	SA: 15.44 ± 1.99	SA (17M; 49F)	Explore the associations between cholesterol and suicidal behaviour	Metabolic: blood serum samples	SA: ↓ cholesterol levels ~ attempted suicide history
		PC: 15.19 ± 1.68	PC (15M; 39F)		Functional: hospital records	
	[199]	SA: 16.8 SEM = .74	SA (3M; 6F)	Investigate platelet PBR density in suicidal teens	Metabolic: blood serum samples	SA: ↓ platelet PBR density
		PC: 16.5 SEM = .5	PC (7M; 3F)		Functional: hospital records, SPI, SRS	
	[155]	SA: 15.87 ± 1.56	SA(10M; 25F)	Evaluate the relationship between plasma serotonin levels and psychometric measures in suicidal adolescents	Metabolic: blood serum samples	SA: ↓ plasma 5-HT level ~ ↑ suicidality. (5-HT did not discriminate between the psychiatric diagnostic categories)
		PC: 16.29 1.81	PC (19M; 11F)		Functional: hospital records, SPI	
		ER: 16.91 2.47	ER (13M; 38F)			
		HC: 15.26 1.41	HC (45M; 50F)			
*Alcohol and substance use*	[200]	BPD-O: 12.9 ± 3.1	BPD-O (77M; 68F)	Investigate obesity in paediatric bipolar patients and notable correlates	Metabolic: BMI	BPD: SUD ~ 2.8 fold increased prevalence of BPD-OB.
		BPD-NO: 13.3 ± 3.0	BPD-NO (108M; 95F)		Functional: K-SADS-P	
*Clinical syndrome*	[162]	MDD: 24.1 ± 3.2	MDD (45M; 44F)	Examine the association between MDD in childhood and BMI in adulthood	Metabolic: BMI	MDD: ↑ BMI at FUP (in adulthood)
		HC: 22.2 ± 2.9	HC (43M; 45F)		Clinical: KSADS,	
	[140]*	MHP: 28.74 ± 10.38	MHP (38M; 28F)	Identify changes in the rates of obesity in never-treated patients with mood disorder over 4 years of follow-up.	Metabolic: BMI	MHP: clinical improvement ~ ↑ BMI
			(40 MDD, 26 BPD)		Clinical: HAMD	

Note. Sample : BPD bipolar disorder, BPD-O bipolar disorder with obesity, BPD-NO bipolar disorder without obesity, ER emergency room patients admitted for suicide attempt, HC healthy controls, FH+ family history of depression, MDD major depression disorder, MHP mental health patients (mixed diagnosis sample), PC psychiatric control (i.e. psychiatric diagnosis but no suicide attempt), PD panic disorder, SA suicide attempters, SUC suicidal tendencies (either ideation, threat or attempt)

Measures : BMI body mass index, BP blood pressure, CRP C-reactive protein, GAF global assessment of functioning, HDRS Hamilton depression rating scale, K-SADS schedule for affective disorders and schizophrenia for school age children, SPI suicide potential interview, SRS suicide risk scale, SSI scale of suicide ideation

Findings : ↑ = Increased, Improved or Higher, ↓ = Decreased, Reduced or Lower, ~ = ‘is associated with’, 5-HT serotonin, FUP follow-up, PBR peripheral-type benzodiazepine receptors

* indicates that the study features more than once in the data synthesis

## Results

A total of 134 studies were included in this systematic review (see Fig. 1); 10 of these studies were featured more than once in the data synthesis to make a total of 144 reported results. As summarised in Table 2, the included studies were categorized according to functional domain in the following proportions: 7.6 % (*k* = 11) investigated social and economic participation, 2.1 % (*k* = 3) physical health, 15.3 % (*k* = 22) suicide and self-harm behaviours, 6.9 % (*k* = 10) alcohol and substance use, and 68.1 % (*k* = 98) clinical syndrome. In regards to neurobiological parameters, 19.4 % (*k* = 28) focused on neuropsychology, 43.1 % (*k* = 62) on neuroimaging, 16 % (*k* = 23) on sleep-wake and circadian biology, 14.6 % (*k* = 21) on neurophysiology and 6.9 % (*k* = 10) on metabolic measures. The range of the mean ages for patient groups across all the included studies was 11.7 to 31.7 years, since those studies that had comparison groups both inside and outside the inclusion criteria of 12 to 30 were still included.

### Neuropsychology

There were 28 studies (a total of 2877 participants; 58.5 % female) that utilised neuropsychology and across these studies 69 % (2037/2877) were patients and 29 % (804/2877) were healthy controls. Among the patient group 47 % (966/2037) had depression, 15 % (301/2037) had bipolar, 12 % (239/2037) had anxiety, and 26 % (531/2037) were classified as other.

#### Functional domains: social and economic participation, physical health, suicide and self-harm & alcohol and substance use

Our systematic search found an association between neuropsychology and three functional domains (i.e. social and economic participation, suicide and self-harm and alcohol and substance use). No studies that met our criteria investigated physical health. The relationship between global deficits in cognition and social and economic participation in mood disorders is unclear as there were only two studies; one reporting a positive relationship [24], and the other no relationship [29]. The former study utilised a mixed psychiatric sample that consisted of mood disorder and psychosis patients, which may have influenced the results given the well-supported relationship between social and economic participation and neuropsychology in patients with psychosis [30]. However, the latter study only investigated MDD (*N* = 16) and utilised the Global Assessment of Functioning scale (GAF) as a measure of participation, which could be problematic as the rating can be made on the basis of symptoms or functioning. Thus, it is clear that more studies are needed in this population to resolve or clarify such findings.

Studies exploring specific neuropsychological capabilities have provided greater insight into how these relate to functional domains that can be ambiguous when investigating global cognition. Of the neuropsychological studies reviewed, executive function appears to be particularly associated with social and economic participation as well as suicide and self-harm behaviours. More specifically, three of the five included studies have shown decision-making and conceptual flexibility impairments to be predictive of suicide and self-harm behaviours [31–33]. Taken together, these studies identify a shared pathophysiology among those who have previously attempted suicide, those who were current suicide attempters and those who were currently self-harming; that is, they all showed characteristic deficits in decision-making and cognitive inflexibility. There is also contrary evidence whereby suicide attempters performed better on the decision making task than depressed patients who hadn’t attempted suicide and healthy controls [34], and there were no neuropsychological differences between self-harmers and non-self-harmers [35]. However, these findings may be attributed to methodological differences and/or a modest sample size, especially since the latter study [35] did not distinguish between current self-harmers and previous self-harmers. The notion that impaired decision making and conceptual flexibility may predispose one to suicidal behaviours is supported by evidence showing neurobiological changes in the areas thought to subserve these functions. Namely, structural and functional dysfunction in the orbitofrontal prefrontal cortex have been identified which imply that suicidal behaviour may be associated with deficits in the attribution of importance to stimuli [36–38], although changes in other regions, such as the dorsolateral prefrontal cortex, have been implicated as well [38].

Similar deficits in executive function, particularly in conceptual flexibility, was associated with impaired social and economic participation [39, 40], whereas studies that investigated verbal learning and memory reported conflicting results regarding social and economic participation. Two studies [24, 41] identified a positive relationship with this functional domain, whilst another two studies did not find any association [29, 39]. Notably, logical memory retention (an index of structured learning and memory) was a common measure identified as significant in the positive studies, but it was not utilised in the other two studies; additional studies of structured learning and memory are needed however the evidence to date suggests that there may be an important role for this particular neuropsychological domain with regards to social and economic participation.

#### Functional domain: clinical syndrome

The association between cognitive function and the clinical syndromes of mood and anxiety disorders in adolescents and young adults has previously been reviewed extensively (see [42, 43]), and some of the findings of such reviews have been reiterated by the present systematic review (see below). Interestingly, it has been reported that impaired verbal memory is significantly associated with depression (not specified as MDD) [44] as well as the development of depressive symptoms, in a community sample [45]. These findings implicate a dysfunction in memory occurring earlier in the course of depressive syndrome development while, poor executive function may be associated with more persistent MDD [46]. In terms of delineating symptom severity, individuals diagnosed with unipolar depression with higher levels of depressive symptoms also show increased (i.e. delayed) choice reaction time [47], lower cognitive control of emotional processing [48], and processing speed deficits [49] suggesting that a broader range of cognitive (including social) measures are also associated with depression and may also be sensitive to the severity of illness.

Similar deficits in cognition have been observed among those with anxiety disorders. Greater Social Anxiety Disorder (SAD) symptom severity was associated with poor executive function, specifically cognitive inflexibility in SAD patients [39]. Most studies have identified cognitive deficits associated with OCD, such as, impaired verbal and visual memory [50], information processing [51, 52], and cognitive flexibility [51–53]. As observed in unipolar depression, increased symptom severity in those with OCD is associated with worse selective attention [52], however this has also not been uniformly reported [50]. Comorbidity has been identified as another factor related to the cognitive deficits in OCD, whereby comorbid depression was associated with executive function deficits in these patients [53]. It is clear that the literature for anxiety disorders is less consistent with regard to the pattern of cognitive deficits and their relationship to anxiety and the severity of illness. The findings regarding GAD and cognition are unsurprisingly similar to the cognitive deficits observed in depression, and provide support for a shared underlying neurobiology for these disorders [54].

Clinical trials utilizing neuropsychological function as an outcome measure have demonstrated that verbal memory improves following: (i) lamotrigine treatment in bipolar patients [55]; and (ii) Transcranial Magnetic Stimulation (TMS) treatment in depressed patients [56]. Furthermore, the improved verbal memory performance was significantly associated with improvements in clinical symptoms of mania and depression, in the former study, and with reductions in hallmark symptoms of depression, in the latter. This adds to the close and complex relationship observed between cognition and mood disorders in young people, and reiterates the need for future research to closely examine the direction of these relationships.

### Neuroimaging

There were 62 studies (a total of 3069 participants; 55.5 % female) that utilised neuroimaging and across these studies 62 % (1894/3069) were patients and 38 % (1175/3069) were healthy controls. Among the patient group 28 % (534/1894) had depression, 27 % (520/1894) had bipolar, 15 % (288/1894) had anxiety, and 29 % (552/1894) were classified as other (i.e. mixed psychiatric samples, ADHD, alcohol use disorders, substance use disorder).

#### Functional domains: social and economic participation, physical health, suicide and self-harm & alcohol and substance use

Results indicate that neuroimaging is a particularly useful modality for investigating suicide and self-harm behaviours as well as alcohol and substance use. In contrast, the utility of neuroimaging for investigating social and economic participation [57] and physical health [58] outcomes is yet to be determined due to a lack of studies exploring these relationships. Moreover, consistent with the findings previously discussed (see 'Neuropsychology') linking poor cognitive flexibility and decision making to suicidal behaviours, reduced Anterior Cingulate Cortex (ACC) volume was associated with a higher number of suicide attempts in patients with comorbid MDD and borderline personality disorder [59]. Furthermore, a study investigating ACC function using fMRI demonstrated that individuals with a suicide attempt history had increased dorsal ACC activity when viewing angry faces, and reduced visual, sensory, prefrontal ACC activity to intense happy and neutral faces compared to both healthy and psychiatric controls [60]. It is suggested that the ACC is an important area involved in attentional control that regulates both cognitive and emotional processes [61]. Structural and functional abnormalities in this area may be indicative of attentional control deficits that affect normal cognitive and emotional processes that are associated with an increased risk for suicidal behaviours in this population. However further evidence for this theory is needed since some fMRI studies could not distinguish between suicide attempters and healthy controls using similar decision making and cognitive flexibility tasks. Psychiatric controls demonstrated increased activity in the ACC during the go-no go task [62] and hippocampus during the Iowa Gambling Task (IGT) [34], compared to suicide attempters who were comparable to healthy controls. It may be that the ACC is associated with attentional control related to the emotive processing that has been linked to suicide rather than the higher cognitive processes investigated in these latter studies.

Alcohol and substance use appears to affect multiple brain structures with most studies indicating that alcohol and substance use is associated with a pattern of reductions in brain volume and impairments in brain function. Cannabis use was investigated by two structural MRI (sMRI) studies where it was associated with reduced total white matter volumes [63], and reduced left fusiform gyrus grey mater volumes [64]. Whilst, the only fMRI study investigating cannabis use reported that lower amygdala reactivity was associated with higher rates of cannabis use in MDD patients [65]. One study [66] investigated alcohol use via sMRI and identified that lower prefrontal cortex white matter and overall grey matter volumes were associated with greater levels of alcohol consumption. Collectively, these findings are mostly consistent with the aforementioned neuropsychological studies (see 'Neuropsychology') and previous evidence regarding the neurobiological effects of alcohol and substance misuse on the structure and function of frontal and temporal brain regions [67].

#### Functional domain: clinical syndrome

From the fifty neuroimaging studies investigating clinical syndrome, multiple regions of interest have been studied using a variety of imaging methods.

##### Structural magnetic resonance imaging (sMRI)

In depressive disorders the majority of studies examining brain structure have focused on frontal and limbic regions with mixed findings; although some promising patterns emerge when the severity and/or clinical course of specific disorders are considered. Reduced ACC volumes [68, 69], and increased ACC thickness [70], have been identified in MDD patients compared to healthy controls. Whilst, no significant association with clinical severity or symptoms was found in these studies, reduced ACC volume was associated with higher borderline personality disorder symptom severity but not depression, in patients diagnosed with comorbid MDD and borderline personality disorder [59]. Some further lines of inquiry provide greater detail about how the severity of the clinical syndrome may influence or be influenced by particular brain structures. Decreased grey matter volume in frontal brain regions were evident in patients with a discrete or persistent affective illness compared to those with attenuated syndromes and healthy controls [71], and MDD patients who experienced more than three untreated depressive episodes had reduced subcallosal gyrus volumes, an ACC sub-division [72]. Collectively, these studies reiterate the relationship between reductions in the ACC and depression, and suggest that greater reductions in the ACC may be associated with more severe illness.

Compared to healthy controls, MDD patients had lower amygdala volumes, and no association with clinical severity or illness duration [73], however MDD patients near the onset of their illness had increased amygdala-hippocampal volume ratios that were associated with higher severity of anxiety, but not depression severity [74]. This is consistent with evidence indicating that larger amygdala volumes in GAD patients are associated with greater symptom severity [75]. This suggests that common forms of depression and anxiety may share similar biological processes and genetic liability [76, 77], yet differ in their phenotypic expression. Although distinct pathophysiology may underlie the development of SAD, since lower amygdala grey matter density was associated with greater disease duration and earlier age of onset in this group [78].

Changes to areas of the brain following a course of treatment can provide valuable insight into the success of treatment and how this may have influenced the course of illness. Compared to healthy controls, treatment naïve patients with OCD had larger thalamic volumes, which normalised following paroxetine treatment. These reductions were also associated with a decrease in OCD symptoms. While, CBT treatment for OCD was not associated with change in thalamic volumes [79], greater symptom improvement following CBT was associated with a normalised metabolism in the ACC [80], and with increased prefrontal grey matter volumes [81]. These studies are consistent with the association between the lower symptom severity and greater prefrontal grey matter volumes [82]. Higher compulsive symptom severity was associated with reduced pituitary gland volume in OCD patients in males, compared to healthy controls [83], and larger corpus callosum area [84]. Collectively these OCD studies seem to indicate that pharmacological treatment for OCD may be particularly useful for targeting deficits in thalamus structure and function, whilst CBT may be better for targeting clinical features associated with prefrontal structures.

##### Functional magnetic resonance imaging (fMRI)

Studies investigating brain function using fMRI have also predominantly focused on frontal and limbic regions. Clinical improvement following lamotrigine treatment for bipolar disorder [85], and SSRI treatment for generalised social phobia [86], were associated with reductions in amygdala activation whilst viewing negative valanced emotional pictures. Similarly, higher activation of the amygdala to emotionally fearful faces compared to happy faces was associated with treatment response to CBT or medication in anxiety patients [87]. Bipolar disorder pharmacotherapy treatment responders compared to non-responders had greater amygdala functional connectivity within the frontolimbic network, and higher amygdala functional connectivity within this network after treatment was associated with greater improvements in mania symptoms [88]. These studies consistently demonstrate a relationship between higher amygdala activity and patterns of treatment response for both bipolar and anxiety disorders. This may be indicative of a shared neurobiological vulnerability for heightened amygdala reactivity associated with stress and the emergence of particular affective disorders [89].

##### Diffusion Tensor Imaging (DTI)

The evidence from these DTI studies collectively indicate that poorer white matter integrity is associated with affective disorders that may be an early marker of disorder. A diagnosis of a depressive disorder, compared to healthy controls was associated with lower fractional anisotropy, a measure indicating poorer white matter integrity, and higher mean and radial diffusivity in the corpus callosum, whilst higher fractional anisotropy and axial diffusivity and lower radial diffusivity in the uncinated fasciculus [90]. Lower fractional anisotropy in the ACC [91], and genu, body and splenium of the corpus callosum as well as the superior and anterior corona radiata [92] is evident in bipolar disorder compared to healthy controls. For those who experienced maltreatment during childhood, compared to healthy controls, MDD at follow up was associated with lower fractional anisotropy in the superior longitudinal fasciculi and the right cingulum-hippocampal projection, whilst substance use disorder at follow up was associated with lower fractional anisotropy in the right cingulum-hippocampal projection [93]. Greater obsession symptom severity in OCD patients was associated with higher fractional anisotropy in the splenium [94]. Lower white matter integrity in the genu of the corpus callosum, anterior thalamic radiation, anterior cingulum and sagittal stratum was associated with higher depression severity in MDD patients [95]. Having a discrete or persistent psychiatric illness was associated with lower fractional anisotropy in the left anterior corona radiata compared to healthy controls, whilst a similar pattern of lower fractional anisotropy within this region was associated with attenuated syndromes of psychiatric illness [96].

##### Magnetic Resonance Spectroscopy (MRS)

In terms of MRS studies, the major metabolites that were investigated include N-acetyl aspartate - a measure of neuronal integrity; choline - involved in cell membrane production, lactate - marks glycolysis has been initiated in an oxygen deficient environment; creatine - indicates metabolism of brain energy; glutamate/glutamine -an excitatory neurotransmitter involved in neural activation; and GABA - an inhibitory neurotransmitter involved in reducing neuronal excitability [97]. Lower total choline in the left striatum was associated with OCD and remained consistent over the course of illness [98]. Compared to healthy controls, bipolar disorder was associated with a higher lactate to N-acetyl aspartate and lactate to creatine ratios [99], and higher N-acetyl aspartate in the ACC and higher N-acetyl aspartate, choline and creatine in the ventral lateral prefrontal cortex [100]. Moreover, bipolar disorder responders had lower left ventral lateral prefrontal cortex glutamate/glutamine [101]. For both MDD patients and healthy controls, higher ACC GABA was associated with lower anhedonia scores, and lower ACC GABA was associated with MDD [68].

### Sleep-wake and circadian biology

This section entails studies that have utilised either sleep physiology (e.g. sleep EEG) and/or sleep-wake monitoring (e.g. actigraphy) or indicators (e.g. cortisol secretion) to determine sleep-wake and circadian function. There were 23 studies (a total of 1609 participants; 59.3 % female) that utilised sleep-wake and circadian biology and across these studies 84 % (1352/1609) were patients and 16 % (257/1609) were healthy controls. Among the patient group 55 % (747/1352) had depression, 3 % (45/1352) had bipolar, 5 % (69/1352) had anxiety, and 36 % (491/1352) were classified as other.

#### Functional domains: social and economic participation, physical health, suicide and self-harm & alcohol and substance use

Sleep-wake and circadian biology appears to be useful for characterising two functional domains in young people, namely social and economic participation, and suicide and self-harm behaviours. Three sleep-wake and circadian studies investigated the relationship between salivary cortisol secretion and social and economic participation. Two of these studies [102, 103] were longitudinal investigations that identified that increased salivary cortisol at baseline [102], and before alprazolam treatment for patients with panic disorder [103] predicted poorer social and economic participation at follow up. Such results suggest that increased HPA activity, indexed by a greater salivary cortisol response, may be indicative of HPA axis deregulation with prognostic significance and not simply a cross sectional marker of stress and active illness. Similarly, the one cross-sectional study [104] found that increased cortisol secretion during a social interaction task was associated with poorer social functioning. Alone, this study would seem to demonstrate that increased cortisol secretion is a state marker of social stress, however in light of the previous longitudinal studies, it is possible that heightened HPA activity is indicative of a persistent dysregulated stress response to social specific cues. Notably, all three of these studies were published over 15 years ago, suggesting the need for new evidence to explore the relationships identified by the longitudinal studies.

Three sleep-wake and circadian studies were longitudinal and identified a relationship with suicide outcomes in MDD patients. At baseline, higher growth hormone secretion during the first 4 h of sleep [105], and higher cortisol secretion in the late hours of sleep [106] were both associated with the emergence of a suicide attempt at follow-up. The final study [107] identified that reduced delta sleep activity was associated with higher levels of suicidality as well as depression severity. Similarly, to the social and economic participation studies, these biological substrates also point to HPA dysregulation as a predictor of later suicide attempts in MDD patient groups.

#### Functional domain: clinical syndrome

Of the eighteen sleep-wake and circadian biology studies, nine studies investigated clinical syndrome utilising cortisol responses. Whilst, the timing of cortisol secretion varied between studies, the findings consistently indicate that increased cortisol response is associated with the development [108–110] and persistence [102, 111] of MDD, whilst remission [112] is associated with reductions in cortisol measures. The heterogeneity of depression becomes clearer with evidence of moderate-to-severe depression being associated with significant blunting of the cortisol response compared to those with mild depression, who had increased cortisol secretion during a stress task [113]. These results suggest that the neurobiological systems mediating the stress response are functioning very differently, and may indicate distinct pathophysiological drivers of depression for these two groups. Specifically, genetically mediated depression associated with increased severity and chronic stress leading to a desensitization of glucocorticoid receptors versus mild to moderate depression arising from predominately environment risk factors with typical HPA abnormalities [113]. This pattern of findings appears to differ for comorbid MDD and anxiety, which was associated with flatter diurnal cortisol slopes (daytime cortisol activity) [114], suggesting that the presence of anxiety influences cortisol function in a way that contrasts to depression alone.

Studies that investigated the relationship between the sleep-wake cycle and clinical syndrome reported quite consistent findings. When compared to unipolar depression, bipolar disorder is associated with delayed onset and lower levels of melatonin secretion [115], as well as increased rates of delayed sleep [116]. Similarly, poor sleep efficiency and lower sleep duration was reported in both unipolar depression [117] and hypomanic individuals [118] compared to healthy controls. Increased depression severity was associated with reduced delta sleep [107], while higher overnight dissipation of slow wave sleep predicted a reduction in depressive symptoms [119]. Similarly, greater high density REM and lower REM latency at baseline was associated with the development of depression at follow in healthy controls [111], whilst another found that greater high density REM was associated with fluoxetine treatment in patients with MDD [120]. Whilst these sleep-wake cycle deficits are evident in those with a full threshold disorders (both unipolar and bipolar disorder), increased rates of delayed sleep are also evident in individuals with either a discrete disorder or an attenuated syndrome compared to individuals with mild symptoms and healthy controls [121]. This indicates that a similar pattern of sleep-wake deficit are also evident in those with subthreshold disorders and highlight that such deficits can be identified earlier in the course of illness.

### Neurophysiology

There were 21 studies (a total of 2034 participants; 69.6 % female) that utilised neurophysiology and across these studies 74 % (1510/2034) were patients and 26 % (524/2034) were healthy controls. Among the patient group 56 % (851/1510) had depression, 9 % (130/1510) had bipolar, 21 % (313/1510) had anxiety, and 14 % (216/2034) were classified as other.

#### Functional domains: social and economic participation, physical health, suicide and self-harm & alcohol and substance use

Evidence from the included studies indicated that neurophysiology may be particularly useful for characterising suicide and self-harm behaviours, and alcohol and substance use. Three separate neurophysiological studies found an association with specific types of suicide and self-harm behaviours. Specifically, lower contingent negative variation was associated with multiple episodes of deliberate self-harm [122], increased posterior EEG alpha asymmetry was associated with suicidal intent and the lethality of suicide attempt, but not depression severity, in suicide attempters [123], and slower reaction times during incentive based decision making tasks was associated with increased frequency for suicide and self-harm behaviours in remitted MDD patients [124]. Together these findings link abnormal brain functions implicated in decision making processes to different types of self-harm and suicidal behaviours [125, 126].

#### Functional domain: clinical syndrome

With regard to the fifteen neurophysiology studies, a number of these examined the relationship between treatment response and neurophysiological markers using a number of methods. Firstly, increased startle response was associated with multiple episodes of depression in depressed individuals [127]. Increased startle response was also associated with the presence of an anxiety disorder compared to healthy controls [128]. In this study, a reduction in acoustic startle response was associated with a reduction in anxiety symptoms following CBT, and a higher startle response at baseline predicted treatment response. Similarly, steep N1 of the Loudness Dependency of Auditory Evoked Potentials (LDAEP) at baseline and higher P2 LDAEP at week 1 predicted anti-depressant treatment response in patients with MDD [129]. These findings are consistent with the phenomenon described in this paper (see 'Neuroimaging') that link maladaptive processes associated with increased threat processing measured at a neural, psychological and behavioural level [130]. These findings implicate the amygdala as the predominate brain region involved in threat processing, however it functions as part of a wider brain circuit involving the dorsal medial prefrontal (anterior cingulate) cortex [131, 132].

Four studies utilised Event Related Negativity (ERN) to characterise the clinical syndrome of OCD patient groups. All four studies found that increased ERN was associated with OCD compared to healthy controls [133–136], however none identified a relationship with symptoms severity, treatment status or medication use. Conversely the N100 ERP had an inverse relationship with symptom severity, whilst both the N100 and P200 had an inverse relationship with illness chronicity [52]. These studies indicate that ERN may be a trait measure associated with OCD independent of symptoms severity, diagnosis and/or treatment effects, while the ERP N100 and P200 may be measures sensitive to illness specific factors (e.g. chronicity).

### Metabolic

There were 10 studies (a total of 1,385 participants; 43 % female) that utilised metabolic measures and across these studies 80 % (1133/1385) were patients and 18 % (252/1385) were healthy controls. Among the patient group 22 % (254/1133) had depression, 35 % (400/1133) had bipolar, 6 % (72/1133) had anxiety, and 36 % (407/1133) were classified as other.

#### Functional domains: social and economic participation, physical health, suicide and self-harm & alcohol and substance use

Three metabolic studies investigated the relationship between suicide and self-harm behaviours and cholesterol. Lower total cholesterol levels were associated with a suicide attempt history [137], and increased severity of suicidal behaviour (e.g. ideations, gestures) among those who were currently suicidal [138], while lower high density lipid cholesterol was associated with higher suicide ideation [139]. Together these findings seem to indicate that lower cholesterol is associated with the spectrum of suicidal behaviours, particularly in males. However, this is an area of contention since higher total cholesterol was associated with current suicidal behaviour versus those not currently suicidal. These findings highlight the complex association between suicidal behaviours and cholesterol, and indicate that its usefulness a biological marker needs further clarification.

Two studies examined the correlates of the risk of obesity, indexed by BMI, among young people with mood disorders. Substance use was associated with an 2.8 fold increased risk of overweight/obesity among bipolar patients and increased BMI was associated with better social and economic participation over a 4-year longitudinal study in mood disorder patients (unipolar and bipolar) [140]. The latter study also reported an increase in the prevalence rates for overweight/obesity, consistent with the finding of the former study. There are a number of illness related factors that could explain this relationship which include a return of normal appetite, medication use, self-modulation of mood by overeating [141] as well as biological factors implicated in mood, and metabolic function and weight maintenance, such as leptin [142] and neurotransmitter abnormalities [143].

#### Functional domain: clinical syndrome

See Table 7 for individual results.

## Discussion

As expected, there is a predominate focus in the literature on clinical syndrome in young patients compared to the other four functional domains (i.e. social and economic participation, physical health, suicide and self-harm behaviours and alcohol and substance use). Whilst the neurobiology of these clinical syndromes have been extensively reviewed previously [131, 144], we provide an overview of these findings. Typically biomarkers of the clinical syndrome alone do not readily provide a complete understanding of disability and the risk factors that put young people at greater risk for a worse illness trajectory [3]. This review demonstrates the use of these biomarkers to investigate multiple functional domains in addition to the clinical syndrome. It is clear that the nature of the relationship between the underlying neurobiology and functional domains is an issue that needs to be resolved in this area. However, overall this review exhibits the usefulness of neurobiological parameters to assess these additional functional domains and identify treatment targets, in addition to the traditional focus on clinical syndrome, to optimise interventions and improve illness trajectories.

### Implications for personalised psychiatry

**Fig. 2:**
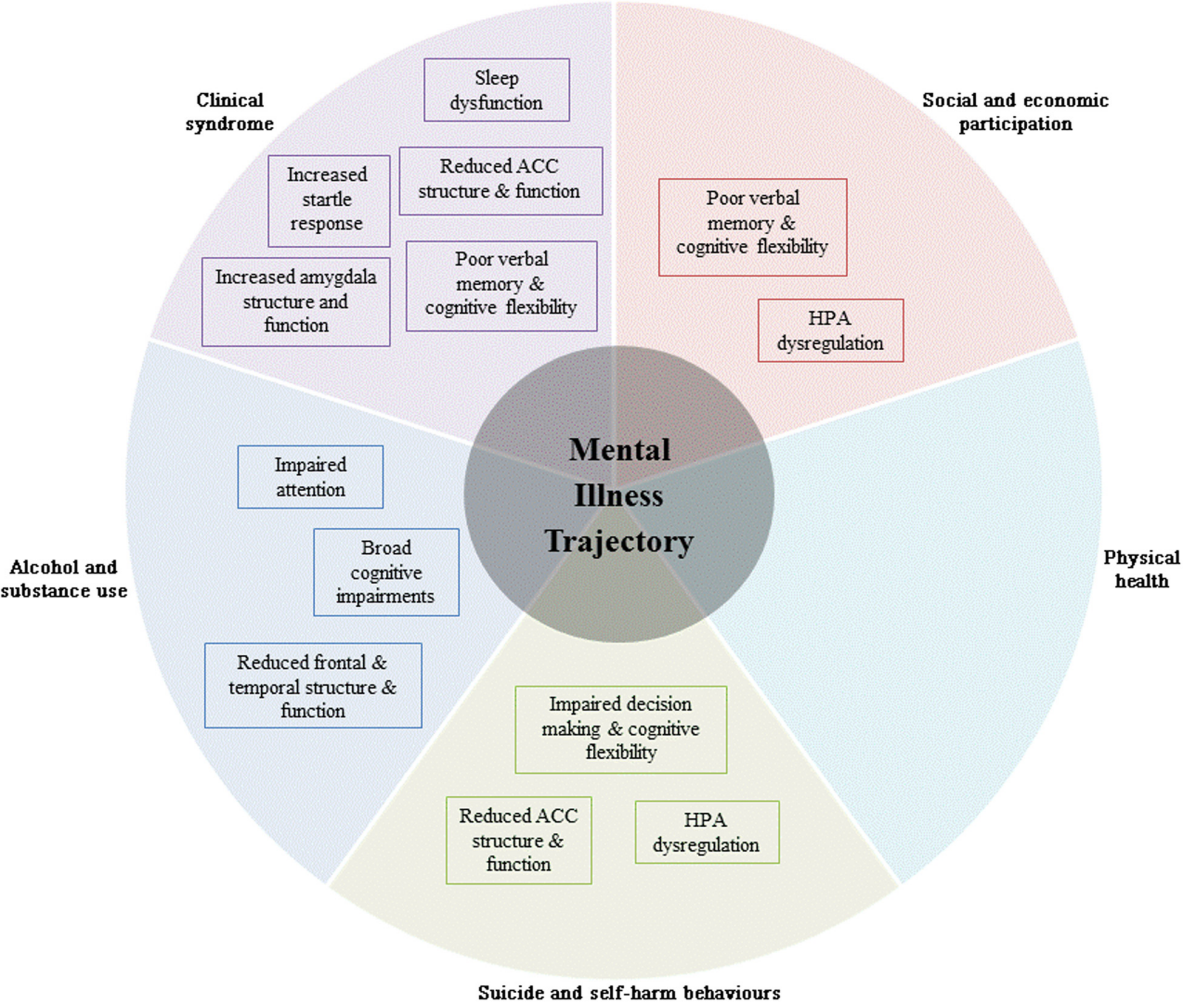
The potential neurobiological targets associated with mental illness trajectory organised by functional domain. This is a model illustrating the potential neurobiological targets for each functional domain identified by this systematic review. At the centre is ‘mental illness trajectory’ that is a term employed here to encompass all the characteristic features of illness, and its associated short term and long term outcomes. Surrounding this are the five functional domains investigated by this review to illustrate how a young person’s mental illness trajectory is made up of and influenced by each of these functional domains. Within the respective sections of the figure, the potential neurobiological targets for each functional domain identified by this systematic review are highlighted in the boxes. The absence of any neurobiological targets in the physical health section of the figure reflects the lack of investigation into this particular functional domain emphasising the need for more research into this area. The clinical syndrome section is the most populated which reflects the predominant focus in the literature on this particular domain. Despite less focus on the remaining three functional domains, the evidence for potential neurobiological targets for these areas is promising for clinical utility and future research

The search for gold standard screening or diagnostic tests has ultimately been unsuccessful, however the increasing emphasis on personalised (or ‘stratified’) psychiatry has the potential to make significant advances in terms of clinical validity and applicability [145]. Whilst, conventional diagnostic methods remain entirely relevant, the addition of the neurobiological markers that indicate prognosis or potential treatment targets are essential for advancing personalised psychiatry. Utilising individual characteristics to guide treatment decisions is key to personalised medicine and providing person-centred care. This review exhibits the utility of the RDoC approach to investigate individual characteristics that extend to functional domains that contribute to ongoing disability and poorer outcomes. By collating the available evidence, this systematic review provides a basis for future investigations to further evaluate the clinical utility of specific neurobiological markers and their relationship with these functional domains (Fig. 2).

#### Social and economic participation

Our findings suggest that those with more severe impairments in memory and executive functioning are at the greatest risk for diminished participation in education, employment and social settings. The use of cognitive tests that characterise memory and executive functions of young people with emerging illnesses may be particularly useful for identifying individuals who are at risk of poor social and economic participation outcomes. Unlike diagnosis, neuropsychological function independently predicts social and economic participation outcomes in young people with emerging mood disorders [146]. The evidence suggests that neuropsychology, specifically memory and executive function, may mediate these outcomes in young people. Therefore, those with identified weaknesses in these areas are likely to require more intensive intervention targeted at these deficits to improve this functional domain. Such treatment practise has demonstrated clinical utility for improving memory performance in adult groups with affective disorders [147]. Moreover, cognitive training was comparable to pharmacological treatment for improving the depression state, whilst also associated with additional benefits such as, no adverse side effects, improved levels of anxiety, and better academic performance [148]. Cognitive training may slow or prevent the impact of cognitive impairment on the social and economic participation domain for young people with an emerging affective disorder.

Notably, the common indicators of social and economic functioning in the reviewed studies varied greatly with some measures (e.g. SOFAS, academic grades) being more useful than others (e.g. GAF) that conflate symptoms and functioning. Of the reviewed studies, there was a lack of focus on social functioning and the relationship with certain neurobiological parameters. The only study to specifically examine this relationship identified that HPA dysregulation, indexed by an enhanced cortisol response during a social task was predictive of social functioning [104]. Although the aforementioned study was cross sectional, longitudinal evidence supporting the predictive validity of HPA dysregulation and greater disability (including social disability), provides further support for the role of HPA dysregulation in social and economic participation [102, 103]. However, it is clear that the role of HPA functioning is complicated given its implications in multiple functional domains including suicide and self-harm behaviours and clinical syndrome.

#### Physical health

Physical health as a functional domain for young people with mood disorders has been notably understudied (only three studies met the inclusion criteria) compared to the other four functional domains. This is not completely unexpected considering that the majority of neurobiological modalities (neuropsychology, neuroimaging, sleep-wake and circadian biology and neurophysiology) are not recognised as being the traditional method for investigating this particular domain [149]. Of note, given that metabolic measures are used to classify physical health outcomes (i.e. BMI is both a physical health outcome and a metabolic measure) it was not deemed appropriate to carry out searches for metabolic measures and physical health outcomes. Future studies may look to improve the methods and/or key terms used to investigate the best available measures to assess and track physical health outcomes in this population.

#### Suicide and self-harm behaviours

Studies investigating suicide and self-harm behaviours spanned across all five neurobiological parameters, and yielded consistent findings across these parameters. Across studies, those with specific executive function impairments in the domains of decision-making and conceptual flexibility appear to be more likely to engage in suicidal thinking or behaviours. Therefore, identifying these deficits may be particularly important for recognising at risk patients and providing effective interventions. Furthermore, our findings provide converging evidence from different measures of brain structure and function that suicide and self-harm behaviours are associated with significant disruptions in decision-making ability. The reduced ACC volumes and activity in the identified neuroimaging studies as well as neurophysiological evidence of lower accuracy at differentiating correct and incorrect responses on a decision-making task corroborate the neuropsychological findings regarding the relationship between suicide and self-harm behaviours and impaired decision making and conceptual flexibility.

Importantly, these findings have major implications for the assessment and intervention of suicide and self-harm behaviours. Firstly, decision making ability should be incorporated into the assessment of young people with mood and anxiety disorders to stratify young people on the basis of risk for suicidal and self-harms behaviours. Whilst, there are certainly other risk factors involved in suicide and self-harm behaviours [150, 151], the findings from this review indicate that decision-making may be a mediator of suicide and self-harm outcomes. Secondly, cognitive remediation interventions aimed at improving decision-making ability in those young people that are identified as having significant impairments may prove to be a successful early intervention to reduce or prevent elevated risk for suicide and self-harm behaviours [152].

The relationship between metabolic studies investigating blood cholesterol and suicide and self-harm behaviours was another major area identified by this review. Three studies investigating cholesterol consistently identified an association between suicide and self-harm behaviours and lower cholesterol levels. Such findings are consistent with evidence that decreased cholesterol levels in the brain may be associated with reduced synaptic plasticity and impaired neurobiological functioning [153]. Furthermore, lower serotoninergic activity has also been associated with reduced cholesterol and implicated in the affective disorders [154], which was evident in another metabolic study identified by this review [155]. It has been suggested that these changes to cholesterol levels may be the result of HPA axis dysfunction, however further studies are needed to explore these associations and investigate the treatment implications.

#### Alcohol and substance use

Converging evidence from neuropsychology, neuroimaging and neurophysiology indicate that alcohol use is associated with global impairments. Unsurprisingly, all the neuroimaging studies suggest that widespread impairment across frontal and temporal areas of the brain are associated with alcohol and substance use. Specifically, reduced brain volume and function are particularly prominent in these areas and these results reflect the findings of neuropsychological and neurophysiology studies that suggest alcohol and substance use to be associated with cognitive impairments and poor attention. Independently each individual study’s limitation of small sample sizes and lack of replication limit their clinical applicability, however together the evidence points to similar phenomena.

An important issue is to determine the how these findings can be used to understand the risk factors associated with alcohol and substance use. Many of the impairments may be the result of alcohol and substance use rather than a mediating factor involved in the risk of engaging in these behaviours. These problems have major implications in terms of assessment and intervention. Whilst, the aforementioned neuroimaging, neuropsychology and neurophysiology studies may be useful for tracking changes in the effects of these behaviours overtime, it is still unclear what assessment measures may be particularly useful in the early identification of individuals at risk for engaging in these harmful behaviours. More specific longitudinal studies before young people engage in alcohol and substance use are needed to differentiate between pre-existing and subsequent effects of alcohol and substance use. These longitudinal investigations will help model the role that risk factors such as risk taking, impulsivity, social occupational factors and decision-making play in the development of poorer alcohol and substance use outcomes.

#### Clinical syndrome

The final functional domain addressed by this review has been extensively reviewed elsewhere [131, 144], and therefore we provide an overview of these findings specific to young people and in the context of the other four functional domains. The primary focus of this particular domain is to identify features of the clinical syndrome that may help characterise a young person’s illness phenotype and/or stage of illness [25, 156]. The majority of studies identified by this review utilised case-control methods to investigate the neurobiological characteristics that separate discrete diagnostic cases from healthy controls, however these distinctions often provide limited clinically useful information. Those studies that sought to describe how specific neurobiological characteristics are related to illness severity (or perhaps more importantly, employed a case-case control method to delineate between cases) were particularly useful for identifying neurobiological or neurocognitive risk factors of poorer illness trajectories. Using a multi parameter approach, this review has been able to collate evidence that covers a number of ‘columns’ in the RDoC matrix (i.e. circuits, physiology, behaviours) to study the pathophysiological drivers of each functional domain, which is crucial for identifying treatment targets [157].

This review has provided evidence to indicate that verbal memory problems may be an early indicator associated with the emergence of depression since these deficits were evident in young people with depression, not identified as MDD, and the emergence of depressive symptoms in a community sample) [44, 45]. However, for those young people with more persistent MDD and more severe depression or anxiety symptoms executive function deficits seem to be more prominent. Similarly, another line of evidence emerging from this review indicates that greater illness severity is associated with greater reductions in the ACC, a prefrontal brain region associated with executive function. Considering the previously discussed results regarding the relationship between executive function, and another two functional domains (i.e. social and economic participation and suicide and self-harm behaviours), this reiterates the role of executive function as a mediating factor that is associated with poorer illness outcomes across multiple functional domains that should be a clear treatment target at both a brain circuit and behaviour level. The benefits of targeting neuropsychological function are made clear by treatment studies that have shown that improved cognitive function following a form of treatment, namely, TMS or pharmacological, has improve hallmark symptoms in both bipolar and depression. Again, this is reiterated by evidence from neuroimaging that treatments increasing ACC function are associated with clinical improvements in depression and bipolar.

The role of comorbid anxiety in depression and bipolar disorder is notable and associated with a substantial increase in morbidity and mortality [158–160]. From a circuitry point of view, the amygdala is one of the primary brain regions in a broader network that is involved in depression and anxiety [131, 132]. Specifically, evidence from this review indicates that increased amygdala volumes are associated with increased anxiety symptom severity in MDD as well as a diagnosis of GAD, reiterating theories that these conditions share similar pathophysiology that cuts across diagnostic boundaries [161]. Importantly, other neuroimaging work utilising fMRI has demonstrated that heightened amygdala activity is associated with both depression and anxiety [132]. This is evidenced by the neurophysiology study identified by this review whereby successful CBT treatment for anxiety disorder was associated with a reduction in anxiety symptoms and the startle response, another index of amygdala reactivity [128]. Together these findings implicate the amygdala as a treatment target that may reduce neurobiological substrates of anxiety, commonly implicated in the emergence of depression and a problematic feature in bipolar disorder. To reiterate the value of amygdala reactivity as a treatment target for anxiety in affective disorders, studies in the present review demonstrated that reduced amygdala activation to negative emotional stimuli, either using pharmacological treatments or CBT, were associated with clinical improvement in both bipolar and anxiety disorders.

From the sleep-wake and circadian biology studies, sleep dysfunction and cortisol secretion have consistently demonstrated a relationship with the clinical syndrome features that allude to differential illness trajectories. For example, sleep dysfunction, characterised by a number of different sleep parameters, was not only associated with the presence of a discrete depressive or bipolar illness, but similar dysfunctions were also evident in those with hypomanic or attenuated syndromes. This is a critical finding since it presents sleep dysfunction as a primary treatment target to prevent illness progression in these affective illnesses. Similarly, increased cortisol secretion was consistently implicated in the emergence of depression [102, 108–110, 112], whilst two metabolic studies suggest that increased BMI is associated with the chronic course of mood disorders [140, 162]. Neurobiologically these findings implicate HPA dysfunction as being a core feature involved in mood disorders, and so interventions aimed at improving the deficits in these brain circuits may be useful to address these clinical outcomes [163].

### Moving towards greater clinical translation

One of the clear problems identified by this systematic review is the lack of consistently used patient groups and assessment measures. For any given functional domain, multiple self-report or clinician rated scales, neuroimaging techniques, and cognitive tests were implemented that often assess the same or similar outcomes, whilst the selection of patient groups varies dramatically for each study. This fundamentally limits the capacity for strong comparisons to be made between studies, or arrive at meaningful and clinically relevant conclusions. For the field of psychiatry to make new ground regarding the underlying neurobiology of psychopathology and its associated outcomes major consolidation of the common standardised measures for these key functional domains and neurobiological parameters should be implemented. Admittedly differences in scientific or clinical motivations will affect the widespread adoption of common measures, however much like the RDoC initiatives focus on key neurobiological domains of interest similar efforts should be made to maximise the standardised measurement of the key functional domains of interest across multiple diagnostic groups in clinical and research settings. In doing this review, we have provided an overview of the essential functional domains and current neurobiological evidence associated with these domains, the next step will be to outline the standard measures that should be drawn upon to promote better consolidation of findings in the psychiatric and neurobiological study of mood and anxiety disorders.

The lack of emphasis on all of these key functional domains and their role in disorder onset, persistence and impact from a publication, reporting and/or research priority point of view is problematic for the clinical translation of psychiatric research. Our study highlights this pertinent issue so that future research may better account for these factors and their relationship to key neurobiological parameters and disorders to improve our understanding of how these functional domains interact or relate to mental illness trajectories.

### Limitations and future directions

Some limitations of this review should be considered. First, few studies investigated a particular functional domain (i.e. physical health) or utilised particular neurobiological parameters, namely sleep-wake and circadian biology or metabolic, which limits the synthesis of these findings and caution is advised when interpreting these results. Secondly, the restricted use of search terms for the functional domains or neurobiological parameters may have limited the identification of key studies, particularly favouring studies reporting current primary disorders rather than lifetime diagnoses. Best efforts were made to be inclusive of as many studies as possible to carry out a complete overview of the literature, however future studies should look to expand on this work by adding key search terms that may have been missed to further the advancement of this growing literature focusing on the functional domains of mood and anxiety disorders in young people. While we focused on RDoC levels of analysis that correspond to ‘circuits’, ‘physiology’, ‘behaviour’, and ‘self-report’, future studies may want to include levels of analysis that include genetics, molecules and cells for a deeper understanding of these neurobiological factors.

Furthermore, future researchers may want to consider including borderline personality disorder into these investigations, given its significant affective component and relationship with these primary mood and anxiety disorders. Limitations associated with the systematic review process should be considered when interpreting the present findings, namely the use of one independent researcher for study selection and lack of a systematic risk of assessment bias since these may have impacted on the reliability and validity of data synthesis. Finally, the wide age range selected for this study is an important caveat that should be addressed in future studies since there are significant developmental/neurobiological changes during this dynamic period of brain development with respect to grey (e.g. pruning) and white (myelination; connectivity) matter processes.

## Conclusions

Mood and anxiety disorders are especially difficult to characterise and treat in young people (age 12 – 30 years) when confounds of normal development and changing environmental influences are prominent. This review identified a predominant focus in the literature on the clinical syndrome, which in our view does not adequately address key individual characteristics, such as suicide and self-harm behaviours or alcohol and substance use, that are involved in disability and persistent illness. Based on the synthesis of results from multiple neurobiological modalities, we provide a detailed summary of how the clinical utility of neurobiological measures may be improved by focussing on personalised assessment of these additional functional outcomes. We suggest that a shift in focus towards characterising the mechanisms that underlie and/or mediate multiple functional domains will optimise personalised interventions and improve illness trajectories.

## Abbreviations

ACC: anterior cingulate cortex
BMI: body mass index
CBT: cognitive behavioural therapy
DLMO: dim-light melatonin onset
DSM: diagnostic and statistical manual of mental disorders
EEG: electroencephalography
ERN: error related negativity
GABA: gamma amino butyric acid
GAF: global assessment functioning
HPA: hypothalamus-pituitary-adrenal
ICD: international classification of diseases
IGT: Iowa gambling task
LDAEP: loudness dependant auditory evoked potential
LICI: long-interval cortical inhibition
MDD: major depressive disorder
MMN: mismatch negativity
MRI: magnetic resonance imaging
MRS: magnetic resonance spectroscopy
OCD: obsessive compulsive disorder
PRISMA: Preferred Reporting Items for Systematic Reviews and Meta-Analyses
RDoC: research domain criteria
REM: rapid eye movement
SAD: social anxiety disorder
SOFAS: social occupational functioning assessment scale
SSRI: selective serotonin reuptake inhibitor
TMS: transcranial magnetic stimulation
